# Simulation of the Interactions of Arginine with Wild-Type GALT Enzyme and the Classic Galactosemia-Related Mutant p.Q188R by a Computational Approach

**DOI:** 10.3390/molecules26196061

**Published:** 2021-10-07

**Authors:** Anna Verdino, Gaetano D’Urso, Carmen Tammone, Bernardina Scafuri, Lucrezia Catapano, Anna Marabotti

**Affiliations:** 1 Department of Chemistry and Biology “A. Zambelli”, University of Salerno, Via Giovanni Paolo II, 132, 84084 Fisciano, SA, Italy; averdino@unisa.it (A.V.); gaetano.durso@univ-rennes1.fr (G.D.); ctammone@unisa.it (C.T.); bscafuri@unisa.it (B.S.); lucrezia.catapano@kcl.ac.uk (L.C.); 2 Interuniversity Center “ELFID—European Laboratory for Food Induced Diseases”, University of Salerno, 84084 Fisciano, SA, Italy

**Keywords:** classic galactosemia, galactose-1-phosphate uridyltransferase, rare diseases, pharmacological chaperones

## Abstract

Classic galactosemia is an inborn error of metabolism associated with mutations that impair the activity and the stability of galactose-1-phosphate uridylyltransferase (GALT), catalyzing the third step in galactose metabolism. To date, no treatments (including dietary galactose deprivation) are able to prevent or alleviate the long-term complications affecting galactosemic patients. Evidence that arginine is able to improve the activity of the human enzyme expressed in a prokaryotic model of classic galactosemia has induced researchers to suppose that this amino acid could act as a pharmacochaperone, but no effects were detected in four galactosemic patients treated with this amino acid. Given that no molecular characterizations of the possible effects of arginine on GALT have been performed, and given that the samples of patients treated with arginine are extremely limited for drawing definitive conclusions at the clinical level, we performed computational simulations in order to predict the interactions (if any) between this amino acid and the enzyme. Our results do not support the possibility that arginine could function as a pharmacochaperone for GALT, but information obtained by this study could be useful for identifying, in the future, possible pharmacochaperones for this enzyme.

## 1. Introduction

Pharmacological chaperones are small molecules that, upon binding to specific target proteins, can stabilize their native conformation, or even correct misfolding in proteins affected by mutations, thus rescuing their original function [1]. Given that many genetic diseases are caused by mutations that can impair the stability of the protein, pharmacological chaperones have been successfully introduced in the therapy for the treatment of several rare diseases, such as lysosomal storage disorders, cystic fibrosis, retinitis pigmentosa, phenylketonuria and Parkinson’s disease [2]. Several known pharmacochaperones act as reversible inhibitors, whereas others are able to bind to allosteric sites and stabilize or select more stable conformations of proteins. Computational approaches such as molecular dynamics (MD) simulations can shed light on the mechanisms of the interactions between the ligands and proteins, and thus play a crucial role in the discovery of suitable molecules [3].

**Figure 1:**
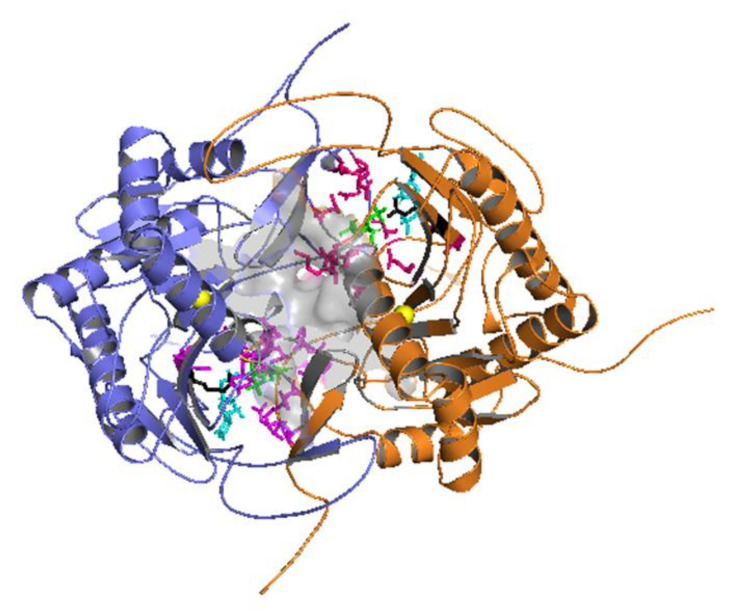
Structure of the model of the homodimeric GALT enzyme carrying the mutation p.Gln188Arg. Chain A is in blue; chain B, in orange. The residues of the two active sites are represented in stick form and colored in magenta, with the mutant residue in black. Galactose-1-phosphate (G1P) is in green, and 5,6-dihydrouridine-5’-monophosphate (H2U) is in cyan. Zn ions are represented as yellow spheres. The surface of the central cavity of the enzyme is represented in grey.

**Scheme 1:**
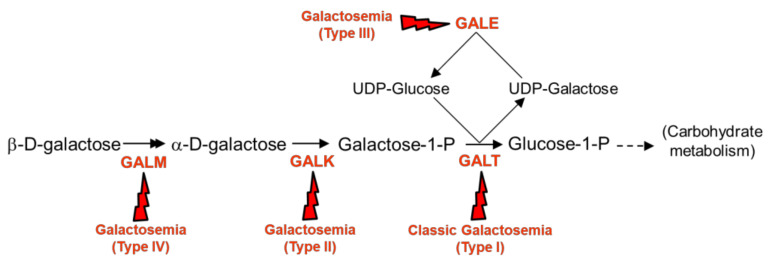
Leloir pathway of galactose metabolism. The enzymes catalyzing the different steps are galactose mutarotase (GALM), galactokinase 1 (GALK), galactose-1-phosphate uridylyltransferase (GALT) and UDP-galactose 4-epimerase (GALE). Different forms of galactosemia derive from the impairment of each enzyme, as represented in the scheme.

Classic galactosemia (OMIM: #230400) is a rare disease affecting people carrying mutations in the gene coding for the enzyme galactose-1-phosphate uridylyltransferase (GALT) (Figure 1), catalyzing the third step in the Leloir pathway of galactose metabolism [4] (Scheme 1).

This disease causes life-threatening symptoms when newborns are exposed to galactose, and severe developmental and neurological complications in late childhood and adolescence even in patients who follow a lifelong galactose-restricted regimen [5]. Since most of the missense mutations affecting GALT are known or predicted to destabilize this protein [6,7], a therapy based on pharmacological chaperones has also been suggested for classic galactosemia [8].

In 2014, Coelho et al. showed that the increased tendency of several GALT mutants to aggregate, associated with protein misfolding, could be a pathogenetic mechanism in classic galactosemia [9]. This led the same group to successfully test the ability of arginine, an amino acid known for its activity as a protein stabilizer with an anti-aggregation effect [10], in improving the activity of GALT mutants, including p.Gln188Arg (the most common mutant enzyme in galactosemic patients), expressed in a prokaryotic model [11]. Despite this success, the administration of arginine to four galactosemic patients homozygous for p.Gln188Arg mutation resulted in no effects. Thus, it was deduced that, at least for people carrying this mutation, arginine has no potential therapeutic role [12]. However, neither study was an in-depth analysis of the molecular interactions between the GALT enzyme and arginine. Moreover, the number of patients recruited in the clinical trial was very small, and the short duration of the study (1 month) was insufficient for evaluating the effects of arginine on long-term clinical outcomes. Furthermore, a single dose (15 g/day) was tested during the clinical trial, and the authors argued that the discrepancy with the results of the prokaryotic model could be due to the higher concentration of arginine used for those experiments [12]. Therefore, further studies would be needed before completely ruling out the possibility of arginine acting as a pharmacochaperone in GALT. In particular, it would be useful to perform a deep characterization of the interactions between this amino acid and the enzyme.

Our research group has been involved for a long time in the characterization of the structural and functional features of the enzymes of galactose metabolism [7,13,14,15,16,17], and in the search for possible therapeutic approaches for classic galactosemia, including the search for inhibitors for the galactokinase (GALK1) enzyme, preceding GALT in the Leloir pathway of galactose metabolism (Scheme 1), in order to reduce the accumulation of galactose-1-phosphate, whose excess is considered the cause for the onset of symptoms specific for GALT deficiency [18,19]. The apparent ability, first, and then the apparent failure of Arg to act as a pharmacochaperone for GALT prompted us to apply computational simulations in order to understand, at the molecular level, the possible interactions between the enzyme and this amino acid, in an effort to predict the putative effect (if any) of arginine on the GALT enzyme.

In this paper, we present the results of our computational approach to investigating the interaction of arginine with the GALT enzyme in different conditions. We hope that this study could shed light on the behavior of the enzyme and can pave the way towards the discovery of a therapy for this disease.

## 2. Results and Discussion

### 2.1. Docking Simulations

A preliminary docking study, performed with a blind approach, showed that arginine tends to interact mainly with the active sites of both wild type GALT (wtGALT) and p.Gln188Arg mutant protein, with a preference for active site A, and additionally with other aspecific sites on the protein surface, without any clusterization (data not shown). Therefore, we considered the active site as a first putative target for the binding of arginine. Targeting the active site of the enzyme could have an effect on the overall stability of the structure of GALT, given that the two active sites of the protein are at the interface between the two subunits forming the quaternary assembly and are formed by residues belonging to both monomeric chains; additionally, the mutant p.Gln188Arg shows a dominant negative effect due to the perturbation of the intersubunit interface caused by the mutation [13,20]. Moreover, there are several examples in which pharmacochaperones are competitive inhibitors of the proteins, such as in the case of migalastat, which is a potent inhibitor of alpha-galactosidase A approved by FDA as a pharmacochaperone in the therapy of Fabry disease [2]. Therefore, as a first approach, we docked arginine in the active site A of both wtGALT and p.Gln188Arg, in the presence or in the absence of the natural substrates, to predict possible effects induced by this amino acid on the enzyme’s quaternary assembly and stability.

Additionally, we also decided to simulate the possibility that arginine could bind to the central cavity of the enzyme, to detect if, in this position, it can influence, either favorably or unfavorably, the activity of the enzyme. The central cavity of the GALT enzyme represents a very interesting target for putative pharmacochaperones [8], and it is formed by residues belonging to both subunits, creating important networks of interactions [7,20]. Thus, we also performed docking simulations targeting the whole central cavity, to investigate if arginine could bind stably and favorably within it, and investigated if this putative interaction could either improve or perturb the structural and dynamic features of the enzyme. Additionally, in this case, we simulated the binding of arginine in the presence and in the absence of both natural substrates in the active site.

The docking results for arginine in the different conditions simulated are reported in Table 1. Figures S1 to S4 show the different poses corresponding to each result and the details of their interactions with the residues of the protein.

The energies of interactions predicted for arginine are negative (favorable) for all the systems, although their absolute values are not that high, indicative of the fact that arginine does not interact strongly with the protein. They are the lowest (most favorable) in those systems in which H2U alone is present in the active site. In these systems, arginine interacts with the residues of the active site (in particular, Arg48 and Lys334, which interact with the negatively charged part of the amino acid, and Glu340, Ser181, and Arg51, which form H-bonds with the polar groups of arginine) and with a strong, favorable interaction with the phosphate group of H2U. When the active site of the enzyme is partly occupied by G1P, the positions of arginine seem slightly different in the two systems. Indeed, in wtGALT, arginine interacts with the residues Glu172, Asn173, and Ser181, the catalytic residue His186, and Gln188; on the contrary, in the mutant p.Gln188Arg, there is an unfavorable interaction with the residue Arg188 that probably displaces arginine towards Arg48, Asn97, and Asp98. In both cases, there is also a favorable interaction with the phosphate group of G1P.

We also simulated the condition in which the active site of the GALT enzyme and of mutant p.Gln188Arg are occupied by both ligands, but as expected, arginine cannot enter in it and stays on the protein surface, contacting a portion of the external part of the enzyme, with a predicted binding energy significantly higher than that obtained in the other simulated conditions (data not shown).

The docking results for the central cavity of the enzymes gave less defined results than those in the active site, because the cavity is very large and, thus, arginine has a higher conformational freedom. However, all the simulations predicted a negative binding energy, suggesting the possibility that arginine could also bind to this cavity. In these systems, arginine frequently binds to Gln38 and Glu40, with occasional contacts with Asp197, Arg201, Thr248, Met341, and Gln344. The predicted binding energies in all these conditions seem not to be significantly different, indicating that neither the mutation nor the presence of the substrate in the active site would affect the binding of arginine in the central cavity.

### 2.2. MD Simulations—Arginine in the Active Site

MD simulations were performed at 310 K (the physiological temperature of the human body) and for 100 ns, a timescale in which it is possible to evaluate if arginine remains or not in the active site. The starting point for the MD simulations was the best docking results, reported in Table 1. Details about the settings of the simulations are reported in the Methods section.

The analyses of the energetic components, of the minimum distance of the periodic images, and of the RMSD of the atom distances for these simulations showed that the systems reached stabilization and that no major perturbation affected them (see Supplementary Files 1 to 6).

**Figure 2:**
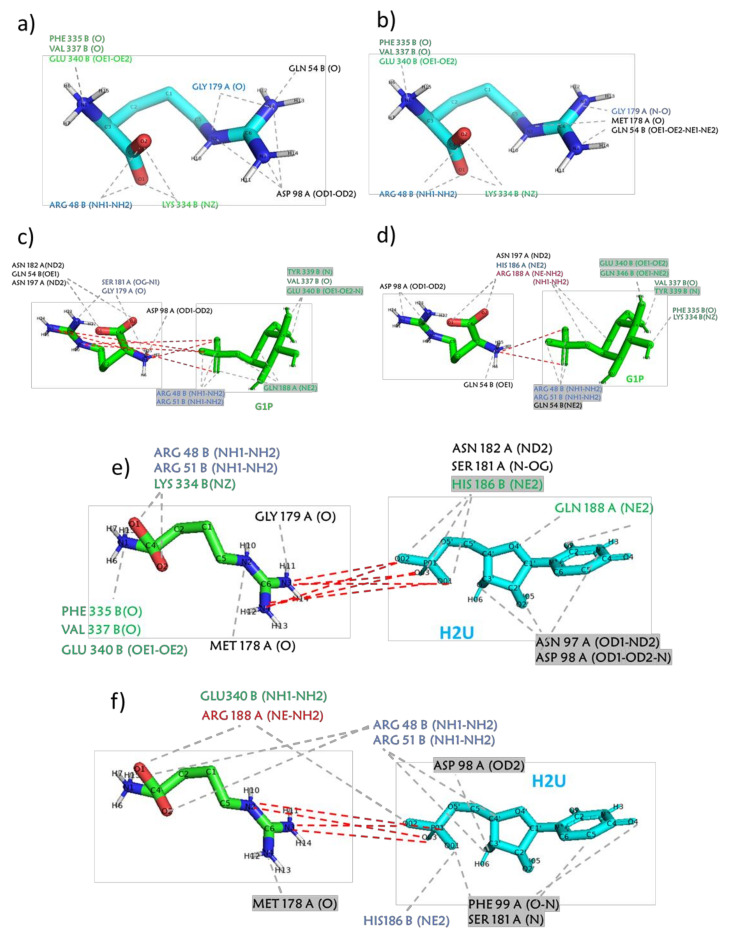
Interactions of arginine in the active site with enzyme and ligands. (**a**) wtGALT + arginine; (**b**) p.Gln188Arg + arginine; (**c**) wtGALT + G1P + arginine; (**d**) p.Gln188Arg + G1P + arginine; (**e**) wtGALT + H2U + arginine; (**f**) p.Gln188Arg + H2U + arginine. Gray background indicates interactions that persisted for more than 50% of the simulation time. Gray dashed lines indicate H-bonds. Red dashed lines indicate interactions between the ligands.

From the data obtained by the two different replicas of the simulations, it appears that arginine is not bound stably to the active sites of both the wild type and the mutant enzyme, irrespective of the absence or the presence of either ligand (Figure S5). When arginine binds into the active site and ligands are not present, arginine occupies the cavity that hosts G1P (the same identified with the docking simulation) and binds to residue Arg48 and to residues belonging to loop 334–340 in both wtGALT and p.Gln188Arg (Figure 2a,b). When G1P alone is in the active site, arginine occupies the place that usually hosts H2U but interacts only with Asp98 in wtGALT, in addition to the phosphate group of G1P itself (Figure 2c). In p.Gln188Arg, the interactions are made with Gln54, His186, and Arg188 (Figure 2d). When H2U alone is in the active site of wtGALT, arginine is hosted again in the cavity of G1P and interacts with the same residues listed above. Additionally, arginine also interacts with the phosphate group of H2U. We observed that the presence of a molecule of arginine in the active site of p.Gln188Arg determines the creation of a cluster of positive charges that perturbs not only the interactions that H2U can keep with the active site, but also the binding of arginine itself.

In all these simulations, G1P and H2U remained stably bound to both wtGALT and p.Gln188Arg (Figure S6). G1P stably interacts with Arg48 and Arg51, and, additionally, with residue 188 and residues 339 and 340, in both wtGALT and p.Gln188Arg (Figure 2c,d). H2U in wtGALT is in contact with Asn97, Asp98, and His186, whereas in p.Gln188Arg, it contacts Arg48, Arg51, and Arg188 (Figure 2e,f). The replacement of Gln188 with Arg is able to perturb the pattern of interactions of the substrate [13].

**Figure 3:**
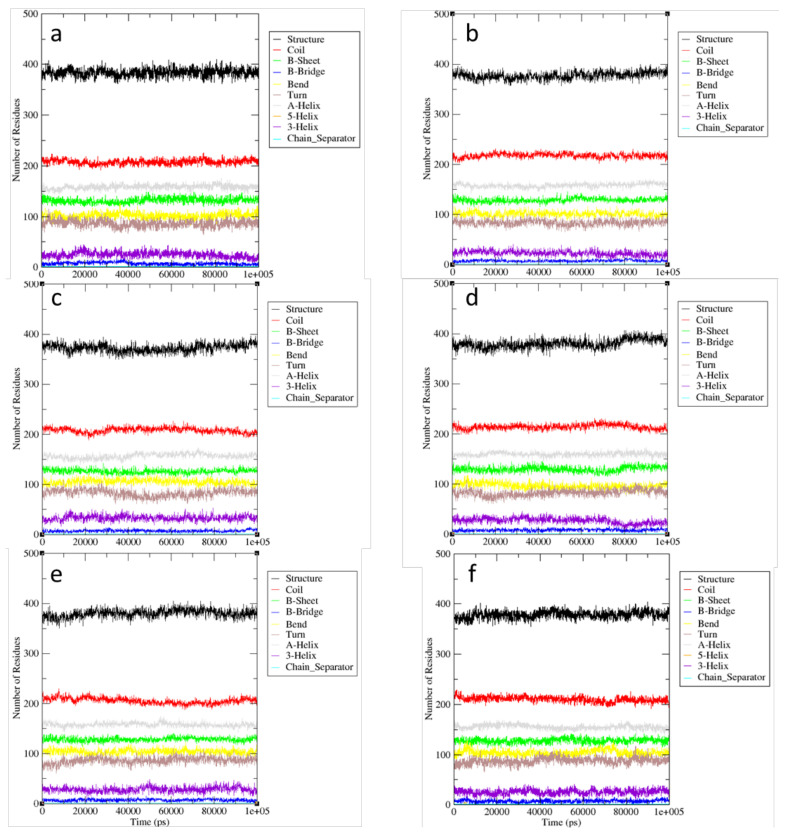
Representative results of DSSP analysis for simulations with arginine in the active site. (**a**) wtGALT + arginine; (**b**) p.Gln188Arg + arginine; (**c**) wtGALT + G1P + arginine; (**d**) p.Gln188Arg + G1P + arginine; (**e**) wtGALT + H2U + arginine; (**f**) p.Gln188Arg + H2U + arginine.

Concerning the global state of the systems, the radius of gyration was constant during these simulations (Figure S7). The solvent accessible surface area (SASA) appears to be affected by the binding of arginine in the active site: this parameter tends to increase when arginine moves away from the active site (Figure S8). The analysis of the evolution of the secondary structures (Figure 3) shows that, in the presence of arginine alone, wtGALT shows a slightly higher presence of irregular structures such as the π-helix with respect to the mutant enzyme. No differences are detectable in both systems in the presence of G1P. In the presence of H2U, irregular structures are detected in the mutant enzyme when arginine is bound to the active site.

**Figure 4:**
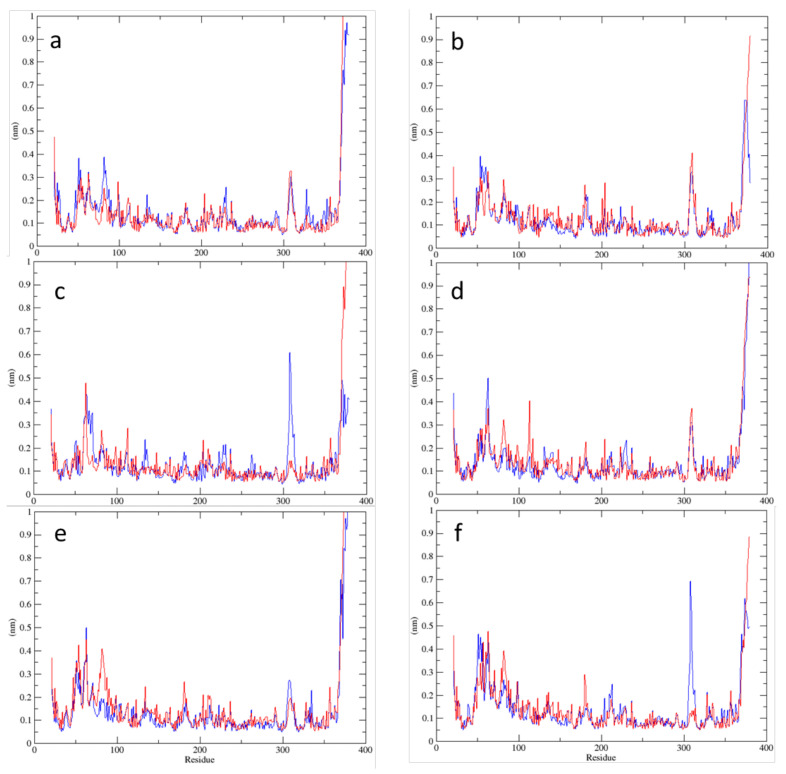
Representative results of RMSF analysis for simulations with arginine in the active site. (**a**) wtGALT + arginine; (**b**) p.Gln188Arg + arginine; (**c**) wtGALT + G1P + arginine; (**d**) p.Gln188Arg + G1P + arginine; (**e**) wtGALT + H2U + arginine; (**f**) p.Gln188Arg + H2U + arginine. Blue lines represent RMSF fluctuation of chain A; red lines, that of chain B.

Comparing the RMSF graphs (Figure 4), the main variability in all the simulations seems to be focused on the same segments already shown in our parallel investigation [17], i.e., mainly segments 50–70 (including segment 50–60 formed by very conserved residues at the intersubunit interface) and 300–320 (a long loop including the conserved residues of the Zn-binding site). For segment 300–320, there is an asymmetrical flexibility of the two chains, more evident in the simulation with G1P for wtGALT and with H2U for p.Gln188Arg.

The analysis of the stable intersubunit interactions is reported for H-bonds in Table 2. Almost half of the stable intersubunit H-bonds monitored were also present in the static models, whereas the others were formed during the simulations. The average number of intersubunit H-bonds per timeframe was very similar in the simulations involving wtGALT with respect to the equivalent simulations involving the mutant enzyme, also considering the variation between the two different replicas of each simulation. This parameter is constantly lower than the same parameter obtained for simulations in the absence of arginine [17]. The most notable difference is visible in the simulations of arginine in the presence of H2U, where the average number of H-bonds per timeframe was higher in wtGALT than in p.Gln188Arg. These data show that arginine does not have a favorable effect on the intersubunit contacts in the mutant enzyme; rather, it seems to perturb the intersubunit interactions in these systems.

The analysis of the stable intersubunit salt bridges during the simulations is reported in Table 3. Only a few stable interactions of this type were present in the systems during the simulations, and most of them involved the residue Asp113 of one chain and Arg228 of the other chain. The presence of arginine in the active site seems not to influence their existence. In contrast with the results obtained for H-bonds, the simulation of arginine in the active site of p.Gln188Arg bound to H2U is the one with the highest number of salt bridges (3), but given this low number of interactions, it is difficult to consider this difference as significant.

From the results of these simulations, it seems that, if arginine binds into the active site of the mutant enzyme, it is not able to counteract the loss of activity; rather, it could even worsen it, as in the case of the simulation of arginine in the active site of the mutant enzyme when H2U is also bound to the site.

### 2.3. MD Simulations—Arginine in the Central Cavity

As for the simulations with arginine in the active site, also for these systems, the starting point for MD simulations was the best docking results, reported in Table 1. The MD simulations were conducted at 310 K, but given the size of the central cavity, we decided for these systems to run 200 ns-long simulations, in order to allow arginine to perform a deeper exploration of the conformational space. All the details about the settings of the simulations are reported in the Methods section.

All the analyses of the energetic components (including the total energy, kinetic energy component, potential energy component, pressure, temperature, volume and density), of the minimum distance of the periodic images, of the root mean square deviation (RMSD), and of the atom distances for these simulations showed that the systems reached stabilization and that no major perturbation affected them (see Supplementary Files 7 to 10).

**Figure 5:**
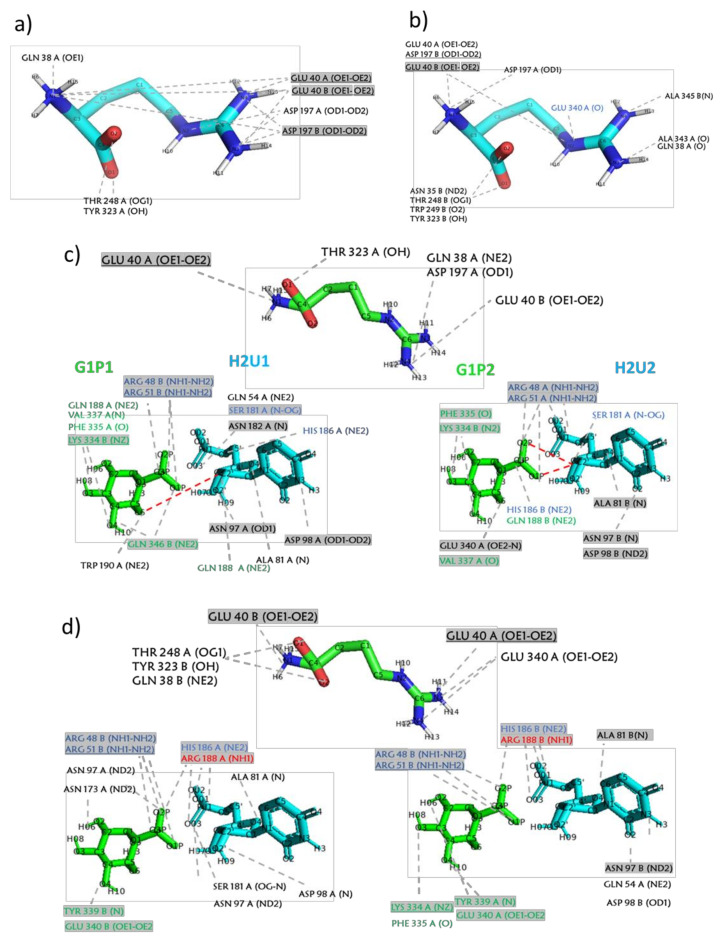
Interactions of arginine in the central cavity with enzyme and ligands. (**a**) Interactions of arginine in the simulation wtGALT + arginine; (**b**) Interactions of arginine in the simulation p.Gln188Arg + arginine; (**c**) Interactions of arginine (top) and of the substrates in the two active sites (**bottom**) in the simulation wtGALT + G1P + H2U + arginine; (**d**) Interactions of arginine (**top**) and of the substrates in the two active sites (bottom) in the simulation p.Gln188Arg + G1P + H2U + arginine. Gray background indicates interactions that persisted for more than 50% of the simulation time. Gray dashed lines indicate H-bonds. Red dashed lines indicate interactions between the ligands.

In all the simulations, arginine stably interacted with the protein, both in the presence and in the absence of the substrates (Figure S9). Additionally, G1P and H2U, in turn, stably interacted with the enzyme for all the simulations (Figure S10). The detailed interactions of arginine and the substrates in these simulations are represented in Figure 5. In the absence of the ligands, as for the docking simulations, arginine mainly interacted with two negatively charged residues belonging to the two protomers of the enzyme, i.e., Glu40 and Asp197, which mainly formed interactions with the guanidinium group of the amino acid. These residues were located in proximity to the Zn-binding site, in a cavity that was putatively identified as an allosteric site for the enzyme [20]. The interaction with these residues seems to be more stable and persistent in wtGALT than in p.Gln188Arg (Figure 5a,b). In the presence of the ligands, arginine remained in stable contact with Glu40 and occasionally interacted with the other residues identified in the docking simulations (Figure 5c,d). G1P maintained H-bonds and salt bridges with the residues Arg48 and Arg51, also seen in the absence of the arginine [17]. Moreover, other interactions were maintained with residues belonging to the flexible loop 335-340. Additionally, it was possible to detect an interaction with Arg188 in the mutant p.Gln188Arg. H2U was mainly bound to Asn97 and Asp98 in wtGALT, and to His186 and Arg188 in p.Gln188Arg. Thus, as seen for the simulations of the GALT enzyme in the absence of arginine [17] and in the previous simulation with arginine bound to the active site, the presence of the mutation is able to perturb the interactions of H2U with the active site residues (Figure 5c,d), but the presence of arginine seems not to be able to modify this situation.

**Figure 6:**
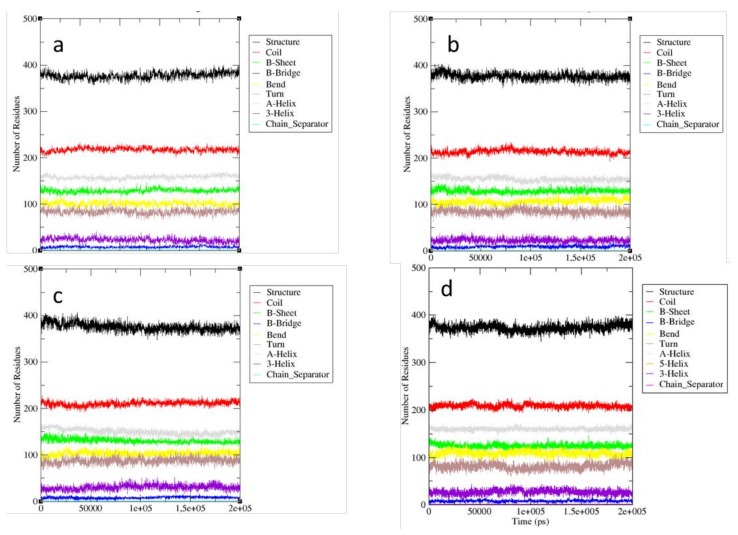
DSSP analysis for simulations with arginine in the central cavity. (**a**) wtGALT + arginine; (**b**) p.Gln188Arg + arginine; (**c**) wtGALT + G1P + H2U + arginine; (**d**) p.Gln188Arg + G1P + H2U + arginine.

**Figure 7:**
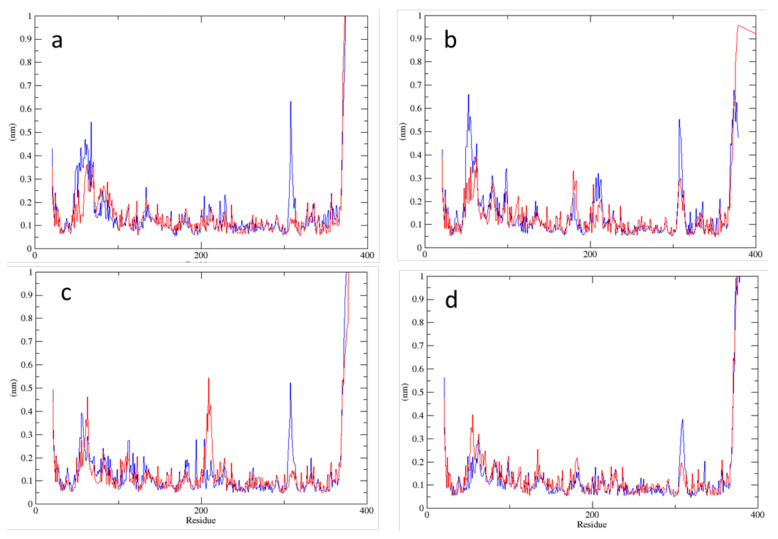
RMSF analysis. Simulations with arginine in the central cavity. (**a**) wtGALT + arginine; (**b**) p.Gln188Arg + arginine; (**c**) wtGALT + G1P + H2U + arginine; (**d**) p.Gln188Arg + G1P + H2U + arginine. Blue lines represent RMSF fluctuation of chain A; red lines, that of chain B.

Concerning the global state of the systems, the radius of gyration was constant throughout the simulations, indicating that the protein did not change its shape (Figure S9). In the presence of arginine, the SASA of both wtGALT and p.Gln188Arg shows a decreasing trend, whereas in the presence of the substrates, wtGALT shows an increasing trend (Figure S10). The analysis of the secondary structures by means of DSSP (Figure 6) showed no significant differences in the presence of arginine only, whereas when ligands were also bound to the active site, there was a higher content of irregular structures such as the π-helix (indicated as a 5-helix in the graph) in the p.Gln188Arg system. Finally, the analysis of the RMSF (Figure 7) showed that, apart from N- and especially C-terminals, the more flexible segments of the protein were those around residues 40, 200 and 320, including portions of the active site. In the presence of arginine only, the mutant p.Gln188Arg showed higher flexibility in the segment around the position of the mutation, whereas, when ligands were also present, the flexibility of the mutant seemed to be decreased, contrarily to what happens to wtGALT. Similarly to previous simulations, in these graphs, it is also possible to detect an asymmetry in the flexibility of the two chains, especially concerning the segment 300–320.

We monitored the variation of stable intersubunit H-bonds in wtGALT and p.Gln188Arg during these simulations. The results are reported in Table 4. Several intersubunit H-bonds that were identified previously in the static models of wtGALT and of p.Gln188Arg [7,20] appeared to be stable, and some of them were conserved in all the different systems. However, as we found in our parallel work [17], several other persistent H-bonds, which were not detectable in the static models, were formed during the simulations and contributed to stabilizing the intersubunit interface. When arginine was present in the central cavity, the average number of H-bonds per timeframe was identical in wtGALT and in p.Gln188Arg, but when ligands were also in the active site, the mutant enzyme showed a notable increase in this parameter. Thus, it seems that arginine bound to the central cavity is able to tighten these interactions between the two subunits.

As for the previous simulations, only a few stable intersubunit salt bridges were detected throughout the simulations (Table 5), and most of them involved the residue Asp113 of one chain and Arg228 of the other chain. In the presence of the ligands, the number of these stable interactions was increased; however, the numbers were so small that they did not allow the evaluation of the significance of these data.

### 2.4. Comparison of the Results of Simulations in the Absence of Arginine

In our parallel work [17], we performed simulations on both wtGALT and p.Gln188Arg, but in the absence of arginine, and we compared the results of those simulations with the ones reported here in the presence of arginine.

When arginine was simulated in the active site, the simulations revealed that this interaction was unstable and that arginine tended to leave the active site during the simulations. When present, it did not favorably affect any structural feature of the enzymes; rather, sometimes, it seemed to perturb them, such as in the case of secondary structures in wtGALT. The mutation-perturbed intersubunit interactions also did not appear to be improved by the presence of arginine in the active site; indeed, the number of both H-bonds and salt bridges was slightly lower in the presence of arginine when compared to those for the corresponding simulations in the absence of this putative pharmacochaperone. Moreover, the presence of arginine in the active site, as could be expected, seemed to perturb the interactions between the enzyme and the substrates. This is more evident in the mutant than in the wtGALT, probably because the accumulation of positive charges in the binding site of p.Gln188Arg determined the formation of a repulsive force that could even result in the expulsion of arginine outside the binding site.

The simulations with arginine in the central cavity showed that the amino acid found a favorable interaction with residues near a putative “allosteric site” [20] and maintained it constantly for the whole simulations. This is interesting, considering that the central cavity of the enzyme is quite big, and that our simulations (both docking and MD simulations) allowed arginine to move freely in this cavity. The presence of arginine in the central cavity did not perturb the secondary structures of the enzyme and seemed to slightly enhance the flexibility of those segments that were in contact with the substrates. This effect, however, was more visible in the absence of the substrates rather than in their presence, and it is difficult to associate this with a functional meaning.

We also analyzed the intersubunit interactions in the simulations in the presence of arginine in the central cavity. When ligands were not present, the average number of H-bonds per timeframe decreased slightly with respect to the simulations in the absence of arginine [17]. When ligands were bound to the enzymes, instead, this parameter decreased in wtGALT and increased in p.Gln188Arg. The number of intersubunit salt bridges was so low that the variations recorded in the different simulations cannot be considered significant.

Finally, looking for a possible effect that the arginine bound in the central cavity could exert on the binding of the substrates in the active site, we noticed that the most stable interactions in wtGALT were maintained with Arg48, Arg51, and residues of the loop 330–340, in addition to the catalytic residue His186. Additionally, in the simulations with arginine in the central cavity, two stable interactions with Asn97, Asp98, and, less frequently, with Ala81, Ser181, and Asn182 were formed. Thus, arginine bound to the central cavity seemed to affect the pattern of interactions of the substrates with the wild-type enzyme. In p.Gln188Arg, however, the stable interactions between the substrates and the enzyme were the same either in the absence or in the presence of arginine in the central cavity. In particular, in all the systems, Arg188 created a strong interaction with H2U, both with H-bonds and salt bridges, and this strong interaction, which persisted for all the simulation time, impaired the mutant enzyme in performing the correct catalysis. In our simulations, the presence of arginine was not able to alter this strong interaction; therefore, we predict that the binding of this amino acid is not able to rescue the enzymatic activity of the mutant enzyme.

## 3. Materials and Methods

### 3.1. Starting Structures

For this work, the starting structures were the models of the human wild-type GALT enzyme and of the mutant p.Gln188Arg, obtained as described previously [7] and based on the crystallographic structure of human GALT obtained by McCorvie et al. [20] (PDB file: 5IN3). Both models contain the ligands galactose-1-phosphate (G1P) and 5,6-dihydrouridine-5′-monophosphate (H2U), which were visible in the crystallographic structure, as well as the Zn ions. The covalent bond between H2U and the residue His186 was not modelled, and the structure of this ligand was corrected in order to restore the normal phosphate group, by using Chimera [21]. This also avoided problems in the parameterization of this anomalous bond in the following steps. The phosphate groups of the ligands were considered in their charged (deprotonated) form throughout the simulations. The structure of the arginine was retrieved from the ZINC database [22]. This amino acid was considered in its zwitterionic form with the side chain protonated throughout the simulations.

The two starting structures of wtGALT and p.Gln188Arg were analyzed with the CASTp 3.0 Web server (http://sts.bioe.uic.edu/castp/calculation.html; last accessed 5 October 2021) [23], which identified three main cavities: the two active sites and the central cavity, with an area of 1772.5 Å^2^ and a volume of 2442.6 Å^3^, formed by 67 residues, of which 32 belong, formally, to the subunit A and 35 to subunit B. These cavities were considered the targets for the following simulations.

### 3.2. Docking Simulations

The binding of arginine to the enzyme was simulated by docking using AutoDock version 4.2, setting up the system with the ADT 1.5.6 software [24]. For all the docking simulations, polar hydrogens were added to the proteins and ligands (except for those groups in ligands that were considered deprotonated), and charges were assigned according to Gasteiger [25].

A grid map with a spacing of 0.375 Å and dimensions of 58 × 80 × 74 points, focused on the residues belonging to the active site of GALT formally identified as “A” (containing His186 of the chain A), as reported in the PDB file, was used to set up the simulations of the binding of arginine into the active site. These simulations were performed by alternatively keeping G1P and H2U in the active site A, and by removing both ligands from the active site A; instead, active site B was left without ligands.

A grid map with the same spacing as above and dimensions of 92 × 112 × 102 points, centered on the central cavity of the enzyme, was used to set up the simulations of the binding of arginine in the central cavity of the enzyme. For these simulations, both ligands were either kept in both binding sites or removed from them.

For each system, 100 docking runs were performed using the AutoDock Lamarckian genetic algorithm, treating the protein as rigid and the ligand as flexible. All the other parameters were kept as default (population size: 150; number of energy evaluations: 2,500,000; and number of generations: 27,000), as is advisable for this molecule with 6 torsional degrees of freedom. The docking poses were clustered using an RMSD value of 2.0 Å. The conformations representative of the best energetic and/or the most populated cluster of poses were selected, saved in .pdb format, and analyzed for their interactions with the enzyme by using DiscoveryStudio (Biovia-DAssaultSystèmes). Those identified as the best compromise for each system (reported in Table 1) were used as a starting point for the following MD simulations.

### 3.3. MD Simulations and Analysis

The simulations were performed at 310 K, corresponding to the normal human body temperature. The simulations with arginine in the active site were performed on three different systems for both wtGALT and the p.Gln188Arg mutant: enzyme + arginine; enzyme + G1P + arginine; enzyme + H2U + arginine. Each of these simulations was performed with 2 replicas, for a total of 12 simulations. The simulations with arginine in the central cavity were performed on two different systems for both wtGALT and the p.Gln188Arg mutant (enzyme + arginine; enzyme + G1P + H2U + arginine).

The MD simulations were performed on two supercomputers available at the CINECA Consortium, the largest Italian computing center (Casalecchio di Reno, Italy): GALILEO, an IBM NeXtScale cluster, with 1022 36-core compute nodes, each containing 2 × 18-core Intel Xeon E5-2697 v4 (Broadwell) at 2.30 GHz, with the Linux Infiniband architecture; MARCONI, a Lenovo NeXtScale Cluster, with 3188 48-core computing nodes, each containing 2x24-core Intel Xeon 8160 (SkyLake) at 2.10 GHz, with the Intel OmniPath architecture. The access to these computational resources was made available thanks to ELIXIR-IT [26]. The package used for the MD simulations was GROMACS 2018.3 [27]. The force field used throughout the simulation was AMBER99SBildn [28,29], and, in order to correctly assign topology and charges to the ligands, the packages ANTECHAMBER [30] and ACPYPE [31] were used according to their instructions. Each of the starting systems was included in a cubic box centered on the protein, with a distance of 1.2 nm from it, filled with water (using the TIP4P model [32]), and neutralized with chloride/sodium ions. The systems were first minimized by applying steepest descent minimization, setting the cut-off for short-range electrostatic and van der Waals interactions to 1.2 nm, and using the grid method to determine the neighbor list. Minimization stopped when the maximum force reached a value lower than 10.0 kJ/mol/nm. Equilibration steps with position-restrained MD simulations (by applying position restraints to all the heavy atoms of the protein to equilibrate the solvent) were run first in NVT (constant number of particles, volume, and temperature) conditions for 100 ps and, subsequently, in NPT (constant number of particles, pressure, and temperature) conditions for 1000 ps. For the NVT equilibration, the V-rescale thermostat [33] was applied; for NPT equilibration, the Berendsen barostat [34] was added to keep the pressure constant at 1.0 bar. At the end of the equilibration, we performed 100 ns-long MD simulations in NPT conditions at a temperature of 310 K for the systems with arginine in the active site, and 200 ns-long MD simulations in the same conditions for the system with arginine in the central cavity. For the production runs, the Berendsen barostat was replaced with the Parrinello-Rahman barostat [35]. The other parameters selected for the production simulations were the leap-frog algorithm [36] for integrating Newton’s equations of motion, the LINear Constraint Solver (LINCS) algorithm [37] to constrain bonds, Verlet [38] as the cutoff scheme in the neighbor searching section, and the Particle Mesh Ewald (PME) method [39] to handle long-range electrostatic interactions.

At the end of the simulations, the trajectories were analyzed using GROMACS analysis tools. The obtained results were plotted by using the XMGrace software (https://plasma-gate.weizmann.ac.il/Grace/; last accessed 5 October 2021). The analysis of the content in secondary structures was performed by using DSSP, the de facto standard algorithm for secondary structure assignment [40]. The percentage of existence of intersubunit hydrogen bonds was calculated with the Perl script plot_hbmap.pl provided by Prof. Justin Lemkul (https://www.thelemkullab.com/; last accessed 5 October 2021), whereas the percentage of existence of salt bridges was determined by using an in-house-developed Perl script on 4000 PDB files extracted from the trajectory using GROMACS commands. We summed up the percentages of existence of hydrogen bonds or salt bridges involving two residues, and only when this sum was equal to or higher than 50 did we consider these two residues as stably interacting during the simulation.

## 4. Conclusions

In the present work, we did not find clear evidence about the ability of arginine to counteract the unfavorable effects of the mutation p.Gln188Arg in the mutant most often associated with classic galactosemia. In particular, the putative binding of arginine to the active site in the mutant enzyme is predicted to create a cluster of positive charges that further destabilizes the quaternary structure, and that, at last, can result in the expulsion of the arginine itself from the site. The putative binding of arginine to the central cavity is predicted to have more favorable effects on the overall structure and function of the enzyme, but also, in this case, we have no clear evidence of a stabilization of the enzymatic structure. Thus, the favorable effect (if any) of arginine on this enzyme is not predicted to be due to an activity similar to that of other pharmacochaperones. Notably, however, arginine is predicted to stably bind to some residues, one of which belongs to a cavity of the enzyme that was previously identified as an allosteric site. This cavity could be considered as a possible target for the development of true pharmacochaperones, also taking into account the interactions identified as crucial in this study and in the other we conducted on this system [17]. This will be the subject of our future studies, in order to find a therapy that can at least alleviate the symptoms of patients suffering from this disabling genetic disease.

## Figures and Tables

**Table 1:** Docking results for arginine used as starting point for MD simulations.

System	Binding Energy of the Representative Pose	Number of Poses in the Cluster	Predicted Interactions with Residues
wtGALT + arginine (active site)	−5.25	35	R48, R51, R333, K334, F335, V337, E340, D348
p.Gln188Arg + arginine (active site)	−5.26	36	R48, R51, R188, R333, K334, F335, V337, E340, D348
wtGALT + G1P + arginine (active site)	−5.55	87	R48, N97, D98, G1P
p.Gln188Arg + G1P + arginine (active site)	−5.22	84	R48, N97, D98, F99, R188, G1P
wtGALT + H2U + arginine (active site)	−6.96	72	R48, R51, G179, S181, K334, F335, V337, E340, Q346, H2U
p.Gln188Arg + H2U + arginine (active site)	−6.76	90	R48, R51, N173, G179, R188, K334, F335, V337, G338, Y339, E340, H2U
wtGALT + arginine (central cavity)	−4.73	26	Q38, E40, D197, R201
p.Gln188Arg + arginine (central cavity)	−5.00	15	Q38, E40, T248, Y323, M341
wtGALT + G1P + H2U + arginine (central cavity)	−5.19	20	Q38, E40, W41, D197, R201
p.Gln188Arg + G1P + H2U + arginine (central cavity)	−5.09	19	Q38, E40, M341, Q344, A345

**Table 2:** Couples of residues involved in stable intersubunit H-bonds in simulations with arginine in the active site. The average number of H-bonds per timeframe is reported for each replica. Dark gray background: intersubunit interactions identified in the initial models and conserved throughout the simulations; bold: stable interactions present in all systems; italics: interactions maintained in the two replicas of each system. Couples of residues are considered to have a stable H-bond interaction when the sum of % of existence of the H-bonds between the two residues is ≥50.

wtGALT + Arginine	p.Gln188Arg + Arginine	wtGALT + G1P + Arginine	p.Gln188Arg + G1P + Arginine	wtGALT + H2U + Arginine	p.Gln188Arg + H2U + Arginine
Average Number of H-Bonds per Timeframe: 26/26	Average Number of H-Bonds per Timeframe: 25/25	Average Number of H-Bonds per Timeframe: 26/22	Average Number of H-Bonds per Timeframe: 24/27	Average Number of H-Bonds per Timeframe: 29/27	Average Number of H-Bonds per Timeframe: 23/26
***ILE32A-LYS120B***	***ILE32A-LYS120B***	***ILE32A-LYS120B***	***ILE32A-LYS120B***	***ILE32A-LYS120B***	***ILE32A-LYS120B***
***ILE32B-LYS120A***	***ILE32B-LYS120A***	***ILE32B-LYS120A***	***ILE32B-LYS120A***	***ILE32B-LYS120A***	***ILE32B-LYS120A***
***TYR34A-GLN118B***	**TYR34A-GLN118B**	***TYR34A-GLN118B***	***TYR34A-GLN118B***	***TYR34A-GLN118B***	***TYR34A-GLN118B***
**TYR34B-GLN118A**	***TYR34B-GLN118A***	***TYR34B-GLN118A***	***TYR34B-GLN118A***	***TYR34B-GLN118A***	***TYR34B-GLN118A***
***ILE198A-ALA343B***	***ILE198A-ALA343B***	***ILE198A-ALA343B***	***ILE198A-ALA343B***	***ILE198A-ALA343B***	***ILE198A-ALA343B***
***ILE198B-ALA343A***	***ILE198B-ALA343A***	***ILE198B-ALA343A***	***ILE198B-ALA343A***	**ILE198B-ALA343A**	***ILE198B-ALA343A***
*ASP197A-GLN344B*	*ASP197A-GLN344B*	*ASP197A-GLN344B*	*ASP197B-GLN344A*	*ARG48A-PHE99B*	*ASP197B-GLN344A*
*ARG48A-PHE99B*	*ASP197B-GLN344A*	*HIS301A-LEU342B*	HIS301A-LEU342B	HIS301B-LEU342A	***TRP41A-ASP197B***
**TRP41A-ASP197B**	ARG48A-PHE99B	**TRP41A-ASP197B**	*HIS301B-LEU342A*	***TRP41A-ASP197B***	***TRP41B-ASP197A***
***TRP41B-ASP197A***	*SER45A-ALA101B*	***TRP41B-ASP197A***	**TRP41A-ASP197B**	***TRP41B-ASP197A***	*ARG228A-ASP113B*
*ARG228A-ASP113B*	HIS47A-PRO100B	***ARG228B-ASP113A***	***TRP41B-ASP197A***	*ARG228A-ASP113B*	***ARG228B-ASP113A***
***ARG228B-ASP113A***	*HIS301B-LEU342A*	GLN30A-ALA101B	***ARG228B-ASP113A***	**ARG228B-ASP113A**	*ARG201B-ASP39A*
*ARG204A-ASP39B*	***TRP41A-ASP197B***	**GLY338A-SER297B**	GLN30A-GLN103B	*GLN30A-GLN103B*	*ARG204A-ASP39B*
*ARG201A-ASP39B*	**TRP41B-ASP197A**	*GLY338B-SER297A*	GLN30A-ALA122B	GLN30A-ALA122B	GLY338B-SER297A
ARG333B-GLU58A	*ARG228A-ASP113B*	GLN56A-GLU58B	*ARG48B-PRO100A*	ARG201B-ASP39A	**GLY338A-SER297B**
*ARG48B-PHE99A*	**ARG228B-ASP113A**	ARG51A-ASP98B	*TYR339A-SER192B*	*GLN103A-GLN30B*	GLN30A-GLN103B
***GLY338A-SER297B***	ARG48B-PRO100A	ARG201A-ASP39B	*SER45B-ALA101A*	ARG201A-ASP39B	SER45A-ALA101B
ALA122A-GLN30B	GLN30A-ALA122B	ARG333A-GLU58B	ARG204A-ASP39B	GLN30B-ALA122A	ARG48A-PHE99B
	GLN30A-GLN103B	*ARG48B-PHE99A*	ARG228A-ASP113B	*ARG48B-PHE99A*	ARG201A-ASP39B
	***GLY338A-SER297B***	SER45B-ALA101A	**GLY338A-SER297B**	ARG51B-ASP98A	GLN30B-GLN103A
	ARG204A-ASP39B	SER45A-ALA101B	HIS114B-GLU220A	***GLY338A-SER297B***	
	GLN169B-ILE32A	GLN103A-GLN30B	ARG201B-GLN38A	SER45A-ALA101B	
		TYR339A-SER192B		SER45B-ALA101A	
		GLN30B-ALA122A		ASP197A-GLN344B	
				ARG204A-ASP39B	
				HIS301A-LEU342B	
				ARG228B-ASP113A	

**Table 3:** Couples of residues involved in stable intersubunit salt bridges in simulations with arginine in the active site. Couples of residues are considered to have a stable salt bridge interaction when the sum of % of existence of the salt bridges between the two residues is ≥ 50.

wtGALT + Arginine	p.Gln188Arg + Arginine	wtGALT + G1P + Arginine	p.Gln188Arg + G1P + Arginine	wtGALT + H2U + Arginine	p.Gln188Arg + H2U + Arginine
GLU58B-ARG333A					
ASP113B-ARG228A	ASP113B-ARG228A	ASP113B-ARG228A		ASP113B-ARG228A	ASP113B-ARG228A
	ASP113A-ARG228B	ASP113A-ARG228B	ASP113A-ARG228B	ASP113A-ARG228B	ASP113A-ARG228B
ARG204A-ASP39B			ARG204A-ASP39B		ASP39A-ARG201B

**Table 4:** Couples of residues involved in stable intersubunit H-bonds in simulations with arginine in the central cavity. Dark gray background: intersubunit interactions identified in the initial models and conserved throughout the simulations; bold: stable interactions present in all systems. Couples of residues are considered to have a stable H-bond interaction when the sum of % of existence of the H-bonds between the two residues is ≥50.

wtGALT + Arginine	p.Gln188Arg + Arginine	wtGALT+ Ligands + Arginine	p.Gln188Arg + Ligands + Arginine
Average Number of H-Bonds per Timeframe: 26	Average Number of H-Bonds per Timeframe: 26	Average Number of H-Bonds per timeframe: 25	Average Number of H-Bonds per Timeframe: 30
**ILE32A-LYS120B**	**ILE32A-LYS120B**	**ILE32A-LYS120B**	**ILE32A-LYS120B**
**ILE32B-LYS120A**	**ILE32B-LYS120A**	**ILE32B-LYS120A**	**ILE32B-LYS120A**
**TYR34A-GLN118B**	**TYR34A-GLN118B**	**TYR34A-GLN118B**	**TYR34A-GLN118B**
**TYR34B-GLN118A**	**TYR34B-GLN118A**	**TYR34B-GLN118A**	**TYR34B-GLN118A**
ILE198A-ALA343B	ILE198B-ALA343A	ILE198B-ALA343A	ILE198B-ALA343A
ASP197A-GLN344B	ASP197B-GLN344A	ILE198A-ALA343B	ILE198A-ALA343B
ARG48A-PHE99B	HIS301A-LEU342B	ASP197B-GLN344A	HIS301B-LEU342A
HIS301A-LEU342B	SER297A-VAL337B	ARG48A-PHE99B	TRP41B-ASP197A
HIS114B-GLU220A	SER45A-ALA101B	HIS301A-LEU342B	ARG201A-ASP39B
HIS47A-PRO100B	**TRP41A-ASP197B**	**TRP41A-ASP197B**	**TRP41A-ASP197B**
GLY338A-SER297B	GLY338A-ASN173B	TRP41B-ASP197A	**ARG228B-ASP113A**
GLN30A-ALA101B	GLN30A-ALA122B	ARG201A-ASP39B	GLN30A-GLN103B
GLN103A-GLN30B	ARG48A-PRO100B	**ARG228B-ASP113A**	GLN30A-ALA122B
GLN30A-GLN103B	ARG204A-ASP39B	SER45B-ALA101A	HIS47A-PHE99B
ARG333A-GLN58B	**ARG228B-ASP113A**	TRP167B-TYR339A	ARG204A-ASP39B
ARG201A-ASP39B	ARG201A-GLU40B	ARG228A-ASP113B	SER297A-VAL337B
**ARG228B-ASP113A**	GLN103A-GLN30B		GLN346A-ALA101B
**TRP41A-ASP197B**	GLN30A-GLN103B		GLN30B-ALA101A
TRP41B-ASP197A	ARG228A-ASP113B		ARG48B-PHE99A
GLY338B-SER297A	ARG201B-ASP39A		ARG51B-ASP98A
ARG48B-PHE99A			ARG201B-ASP39A
GLN224A-HIS114B			ARG228A-ASP113B
			ARG333B-GLU58A

**Table 5:** Couples of residues involved in stable intersubunit salt bridges in simulations with arginine in the central cavity. Couples of residues are considered to have a stable salt bridge interaction when the sum of % of existence of the salt bridges between the two residues is ≥ 50.

wtGALT + Arginine	p.Gln188Arg + Arginine	wtGALT + Ligands + Arginine	p.Gln188Arg + Ligands + Arginine
GLU58B-ARG333A			
	ASP113B-ARG228A	ASP113B-ARG228A	ASP113B-ARG228A
	ASP113A-ARG228B	ASP113A-ARG228B	ASP113A-ARG228B
		ASP39B-ARG201A	ASP39B-ARG201A

## Data Availability

The data are contained within the article or supplementary material.

## Supplementary Materials

Figure S1: Docking results, simulations with arginine in the active site: position of the selected pose; Figure S2: Docking results, simulations with arginine in the active site: interactions; Figure S3: Docking results, simulations with arginine in the central cavity: position of the selected pose; Figure S4: Docking results, simulations with arginine in the central cavity: interactions; Figure S5: Pair distance between enzyme and arginine in simulations with arginine in the active site; Figure S6: Pair distance between enzyme and substrates in simulations with arginine in the active site; Figure S7: Radius of gyration in simulations with arginine in the active site; Figure S8: SASA in simulations with arginine in the active site; Figure S9: Pair distance between enzyme and arginine in simulations with arginine in the central cavity; Figure S10: Pair distance between enzyme and substrates in simulations with arginine in the central cavity; Figure S11: Radius of gyration in simulations with arginine in the central cavity; Figure S12: SASA in simulations with arginine in the central cavity; File S1: Quality check for wtGALT + Arg (active site); File S2: Quality check for wtGALT + G1P + Arg (active site); File S3: Quality check for wtGALT + H2U + Arg (active site); File S4: Quality check for p.Gln188Arg + Arg (active site); File S5: Quality check for p.Gln188Arg + G1P + Arg (active site); File S6: Quality check for p.Gln188Arg + H2U + Arg (active site); File S7: Quality check for wtGALT + Arg (central cavity); File S8: Quality check for wtGALT + ligands + Arg (central cavity); File S9: Quality check for p.Gln188Arg + Arg (central cavity); File S10: Quality check for p.Gln188Arg + ligands + Arg (central cavity).
