# Supplementary material for: Simulation of the Interactions of Arginine with Wild-Type GALT Enzyme and the Classic Galactosemia-Related Mutant p.Q188R by a Computational Approach
Source: Molecules. 2021 Oct 7;26(19):6061. doi: 10.3390/molecules26196061 (PMC8513022; doi:10.3390/molecules26196061)
Supplement: Supplementary file 1 [file molecules-26-06061-s001.zip › supplFigures-siArg_sito+cavita-rev.pdf]

Figure S1

Docking results

Simulations with arginine in the active site

Position of the selected pose

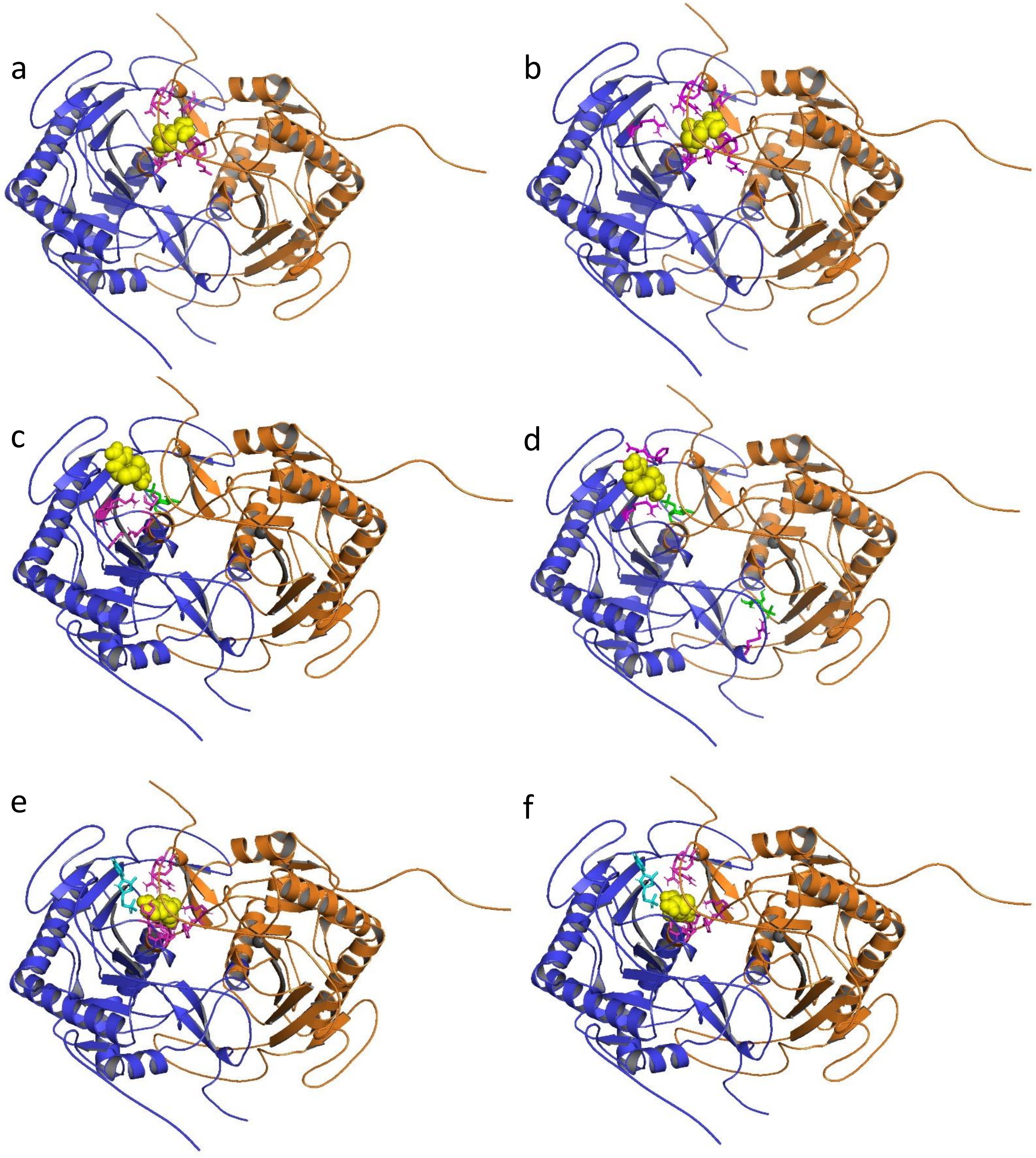

a): wtGALT+arginine; b): p.Gln188Arg+arginine; c) wtGALT+G1P+arginine; d) p.Gln188Arg+G1P+arginine; e) wtGALT+H2U+arginine; f) p.Gln188Arg+H2U+arginine. Arginine is represented in yellow spheres. Color codes as for Figure 1

Figure S2

Docking results

Simulations with arginine in the active site - interactions

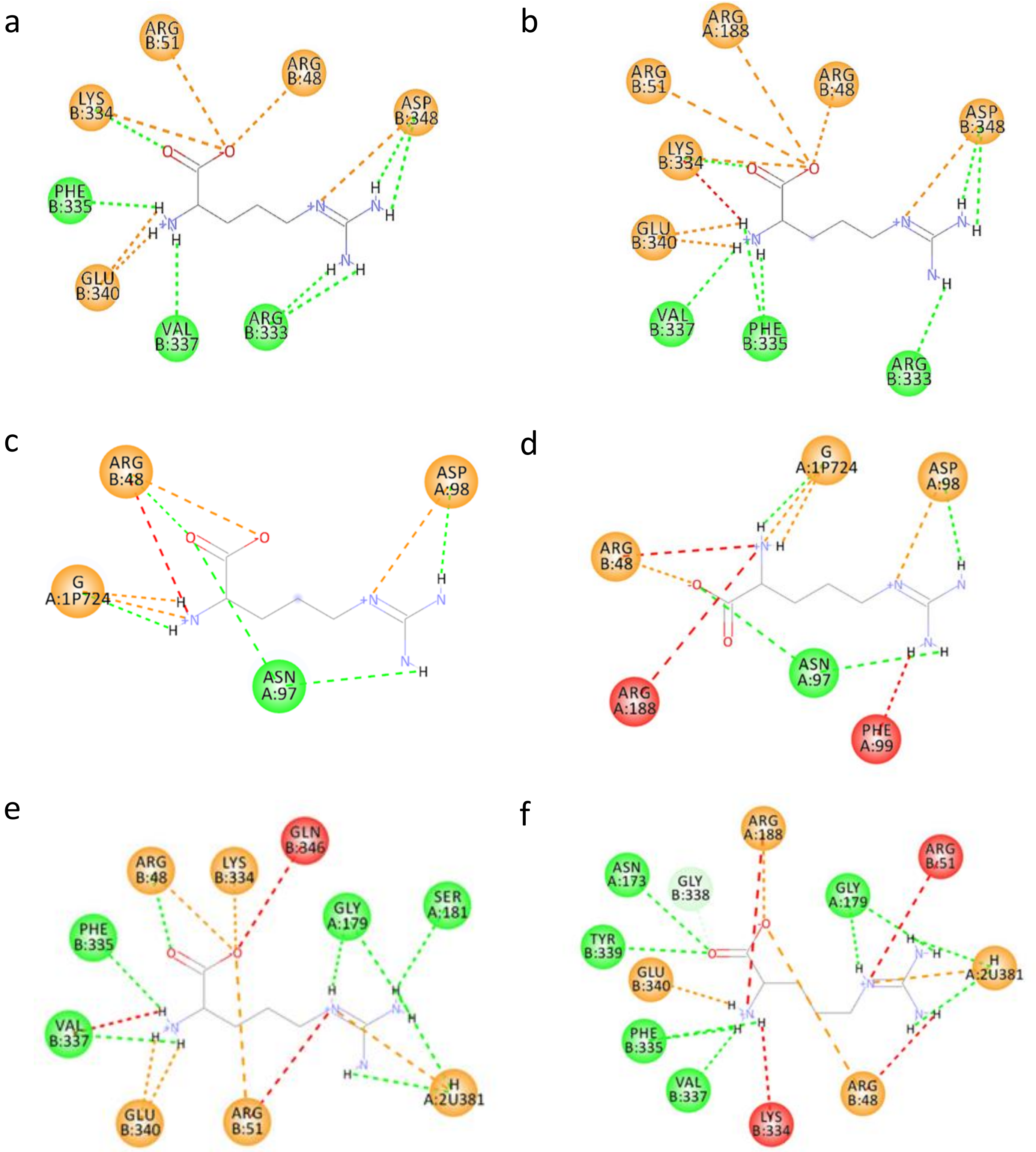

a): wtGALT+arginine; b): p.Gln188Arg+arginine; c) wtGALT+G1P+arginine; d) p.Gln188Arg+G1P+arginine; e) wtGALT+H2U+arginine; f) p.Gln188Arg+H2U+arginine. Color codes: orange: attractive charge; green: hydrogen bonds; grey: other favorable bonds; red: unfavorable bonds.

Figure S3
Docking results
Simulations with arginine in the central cavity
Position of the selected pose

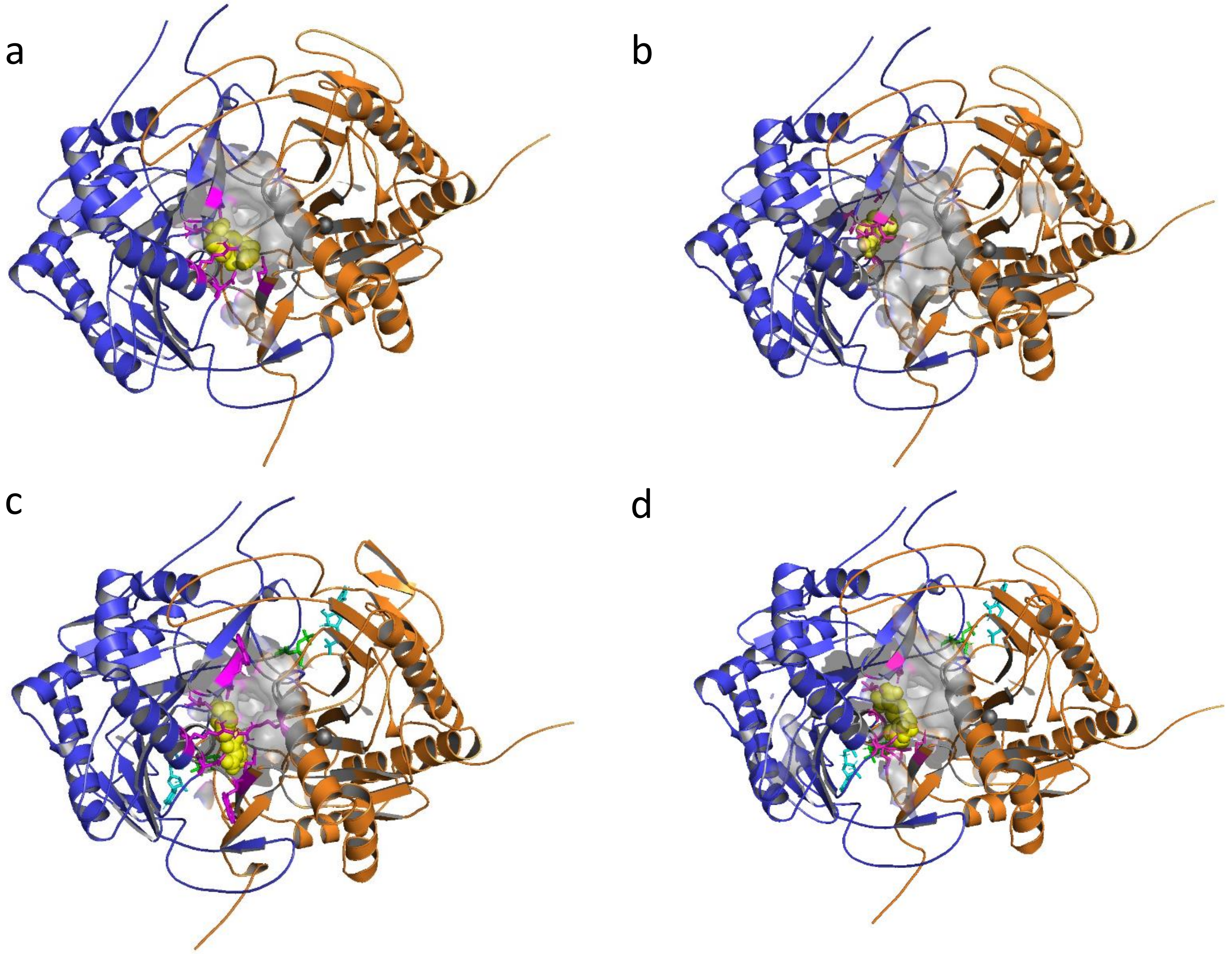

a): wtGALT+arginine; b): p.Gln188Arg+arginine; c) wtGALT+G1P+H2U+arginine; d) p.Gln188Arg+G1P+H2U+arginine. Arginine is represented in yellow spheres. Color codes as for Figure 1

Figure S4
Docking results
Simulations with arginine in the central cavity - interactions

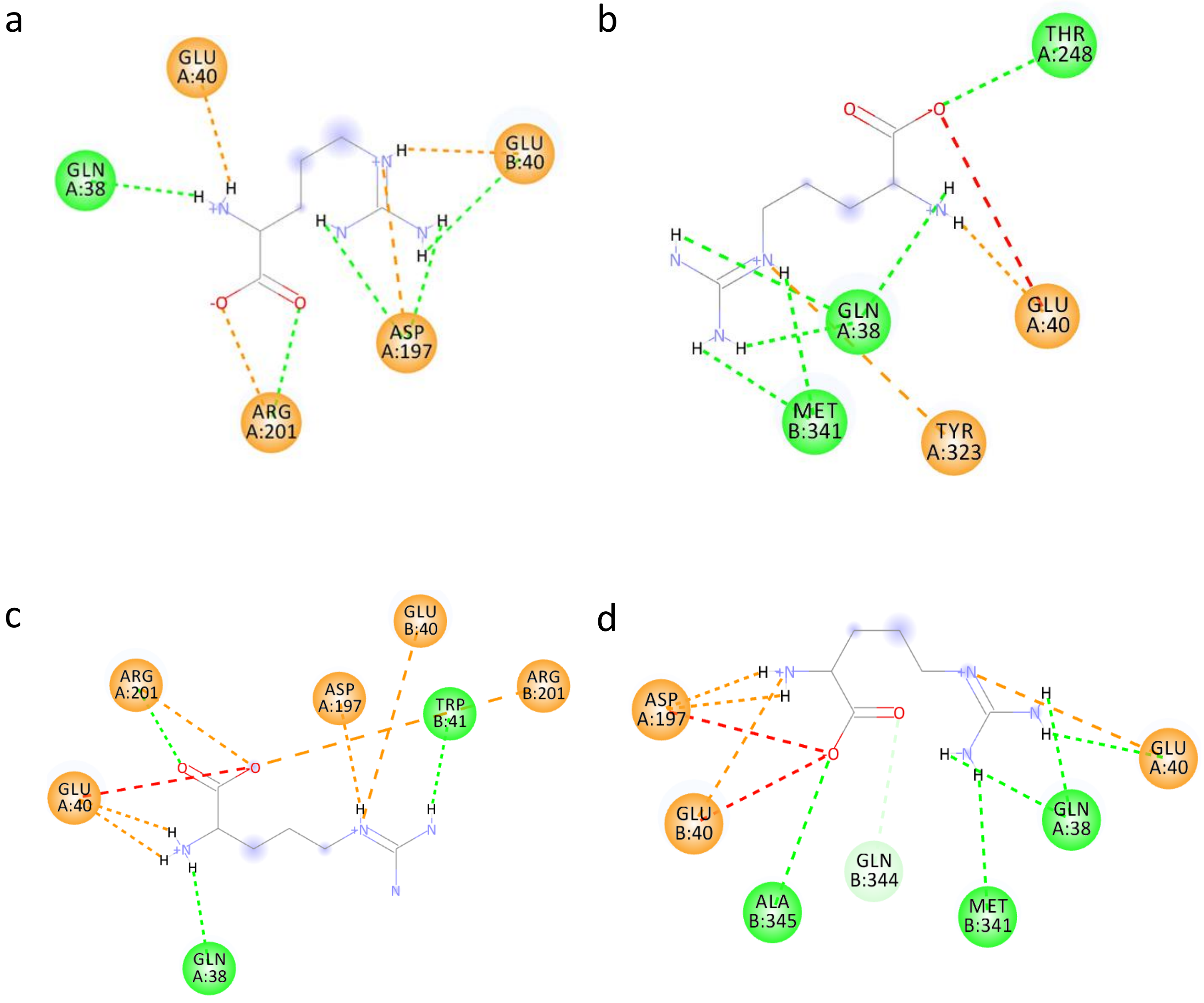

a): wtGALT+arginine; b): p.Gln188Arg+arginine; c) wtGALT+G1P+H2U+arginine; d) p.Gln188Arg+G1P+H2U+arginine. Color codes: orange: attractive charge; green: hydrogen bonds; grey: other favorable bonds; red: unfavorable bonds.

Figure S5
Pair distance between enzyme and arginine, simulations with arginine in the active site

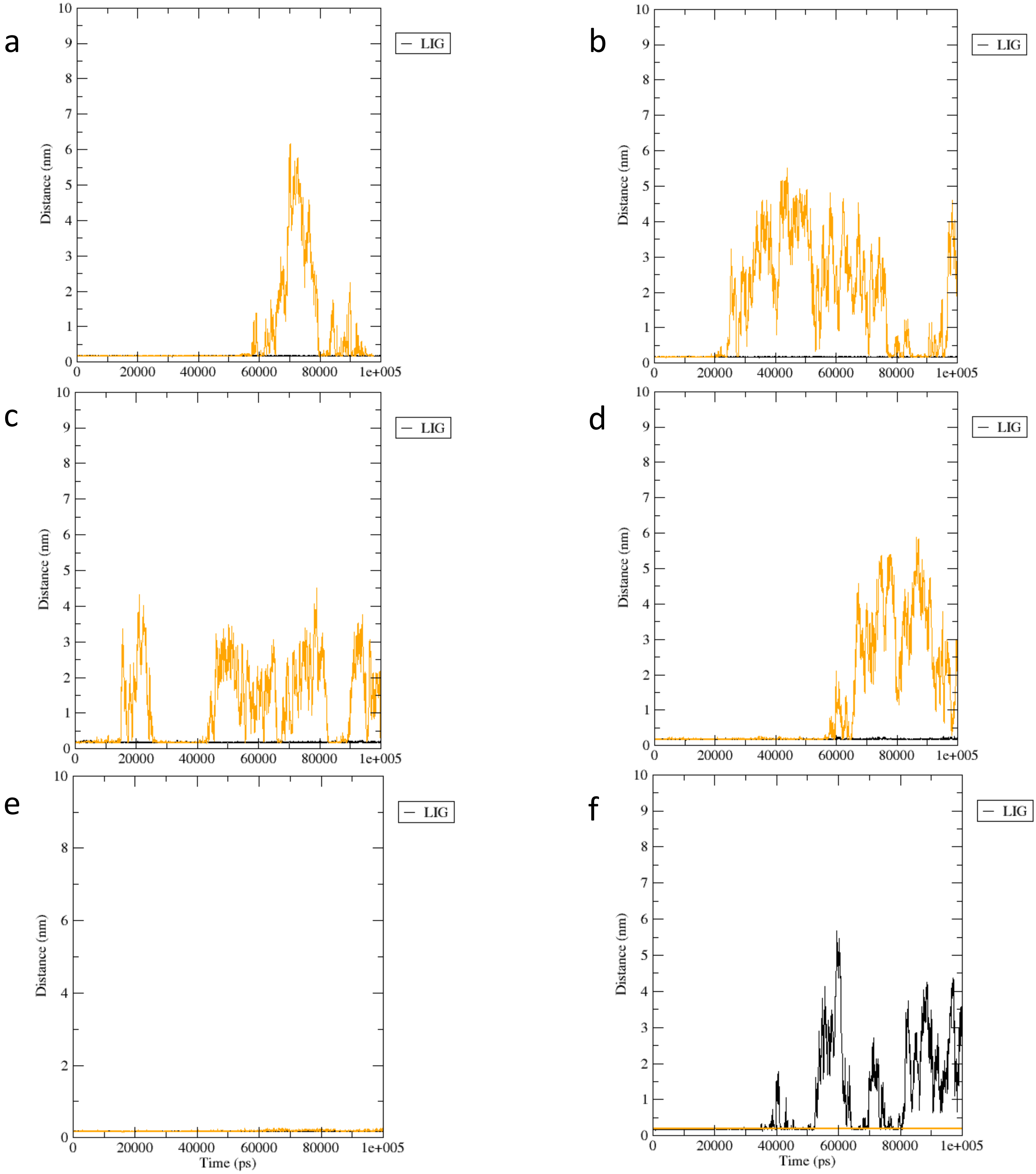

a): wtGALT+arginine; b): p.Gln188Arg+arginine; c) wtGALT+G1P+arginine; d) p.Gln188Arg+G1P+arginine; e) wtGALT+H2U+arginine; f) p.Gln188Arg+H2U+arginine. Black line: first simulation; orange line: second simulation.

Figure S6
Pair distance between enzyme and substrates, simulations with arginine in the active site

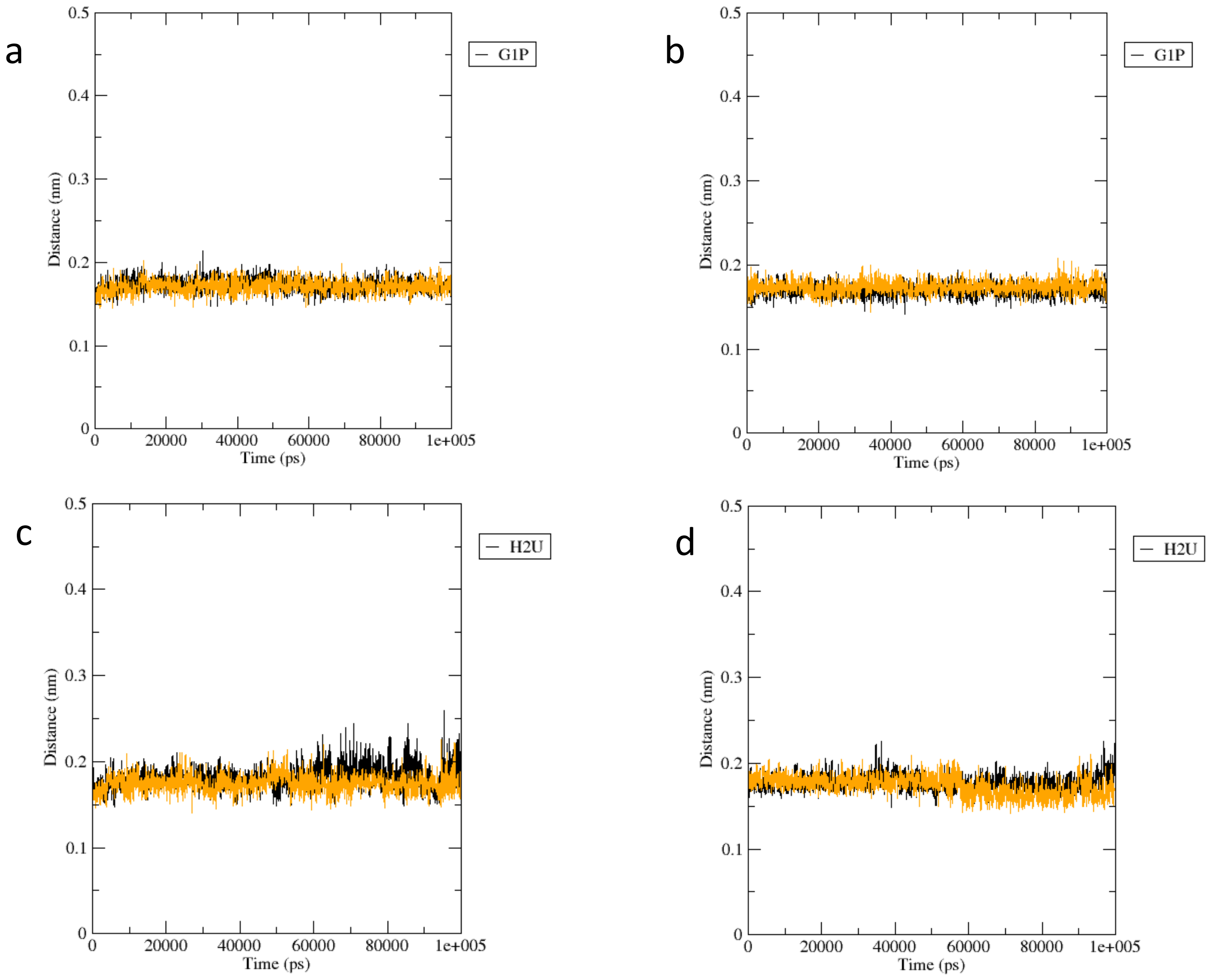

a): pair distance of G1P in the simulation wtGALT+G1P+arginine; b) pair distance of G1P in the simulation p.Gln188Arg+G1P+arginine; c): pair distance of H2U in the simulation wtGALT+H2U+arginine; b) pair distance of H2U in the simulation p.Gln188Arg+H2U+arginine. Black line: first simulation; orange line: second simulation.

Figure S7
Radius of gyration
simulations with arginine in the active site

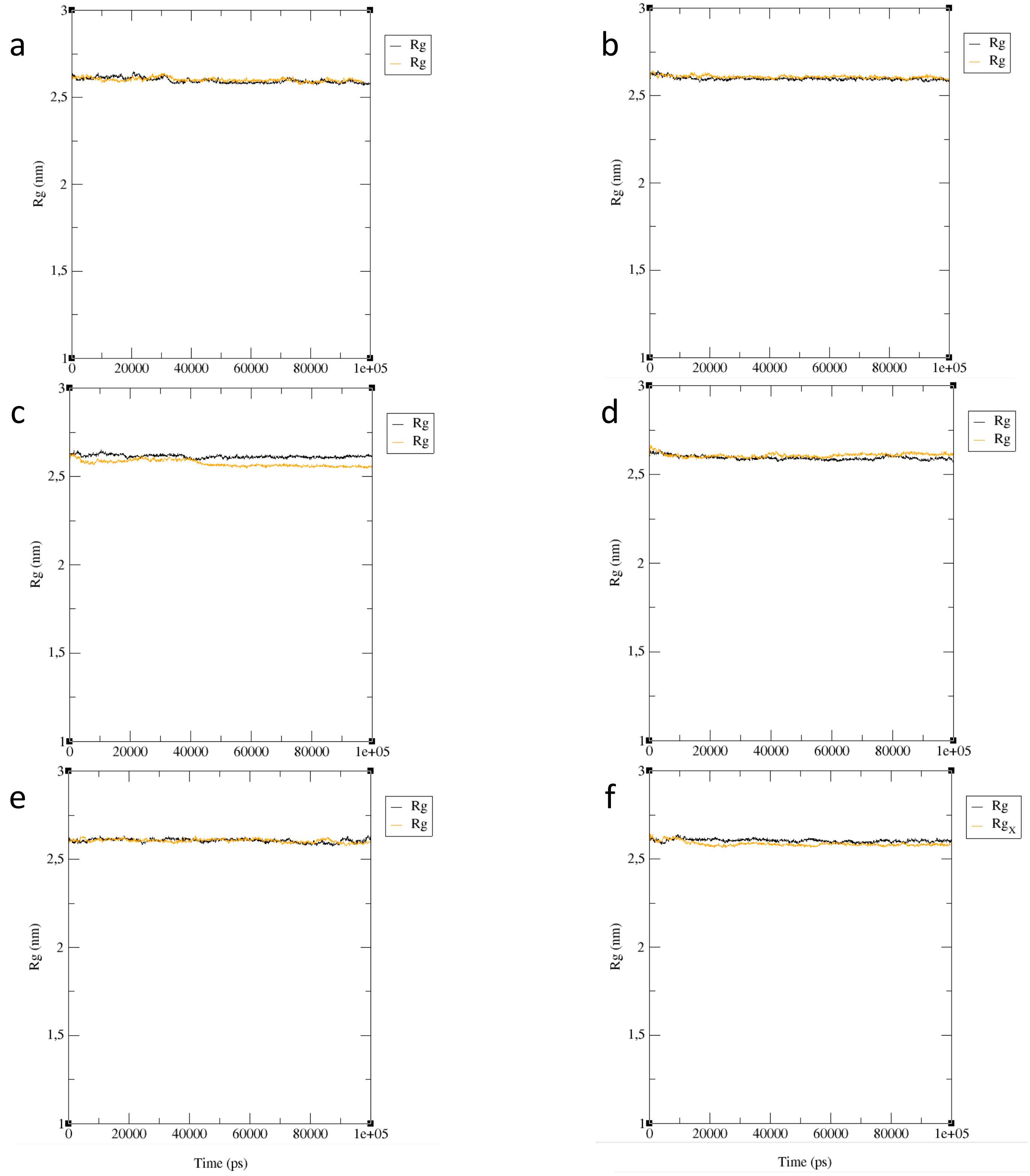

a): wtGALT+arginine; b): p.Gln188Arg+arginine; c) wtGALT+G1P+arginine; d) p.Gln188Arg+G1P+arginine; e) wtGALT+H2U+arginine; f) p.Gln188Arg+H2U+arginine. Black line: first simulation; orange line: second simulation.

Figure S8
SASA
simulations with arginine in the active site

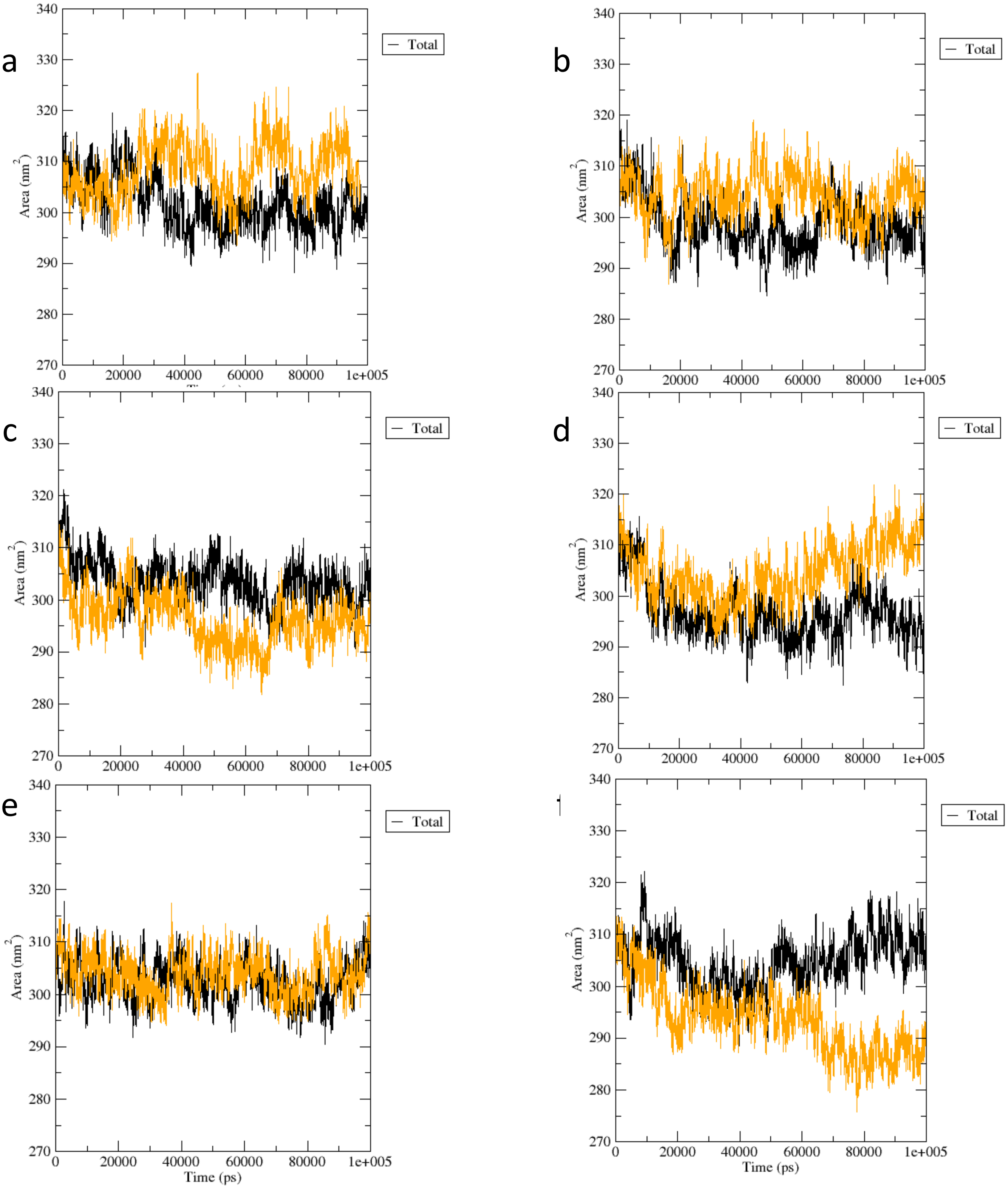

a): wtGALT+arginine; b): p.Gln188Arg+arginine; c) wtGALT+G1P+arginine; d) p.Gln188Arg+G1P+arginine; e) wtGALT+H2U+arginine; f) p.Gln188Arg+H2U+arginine. Black line: first simulation; orange line: second simulation.

Figure S9
Pair distance between enzyme and arginine simulations with arginine in the central cavity

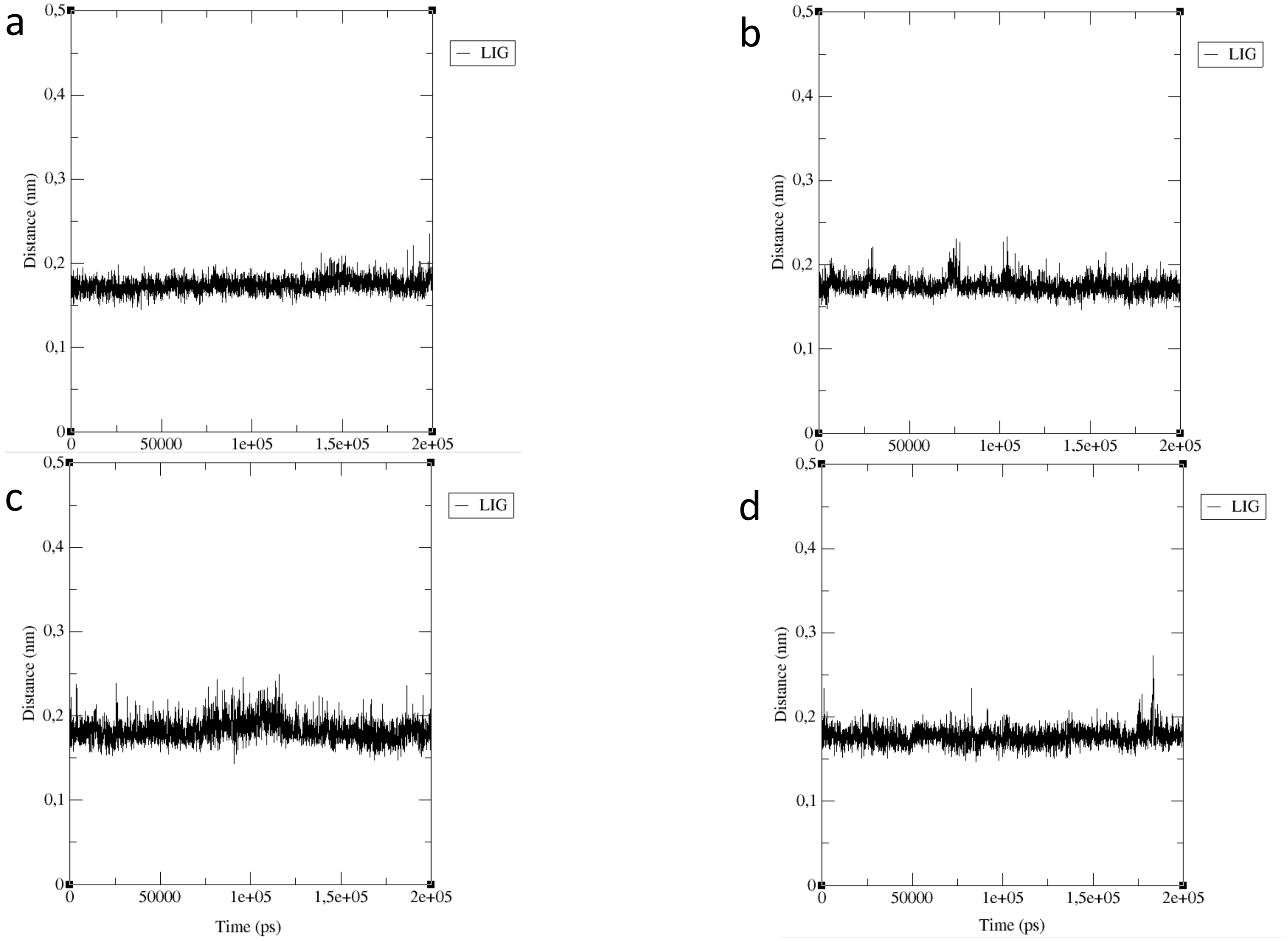

a): wtGALT+arginine; b): p.Gln188Arg+arginine; c) wtGALT+G1P+H2U+arginine; d) p.Gln188Arg+G1P+H2U+arginine.

Figure S10
Pair distance between enzyme and substrates, simulations with arginine in the central cavity

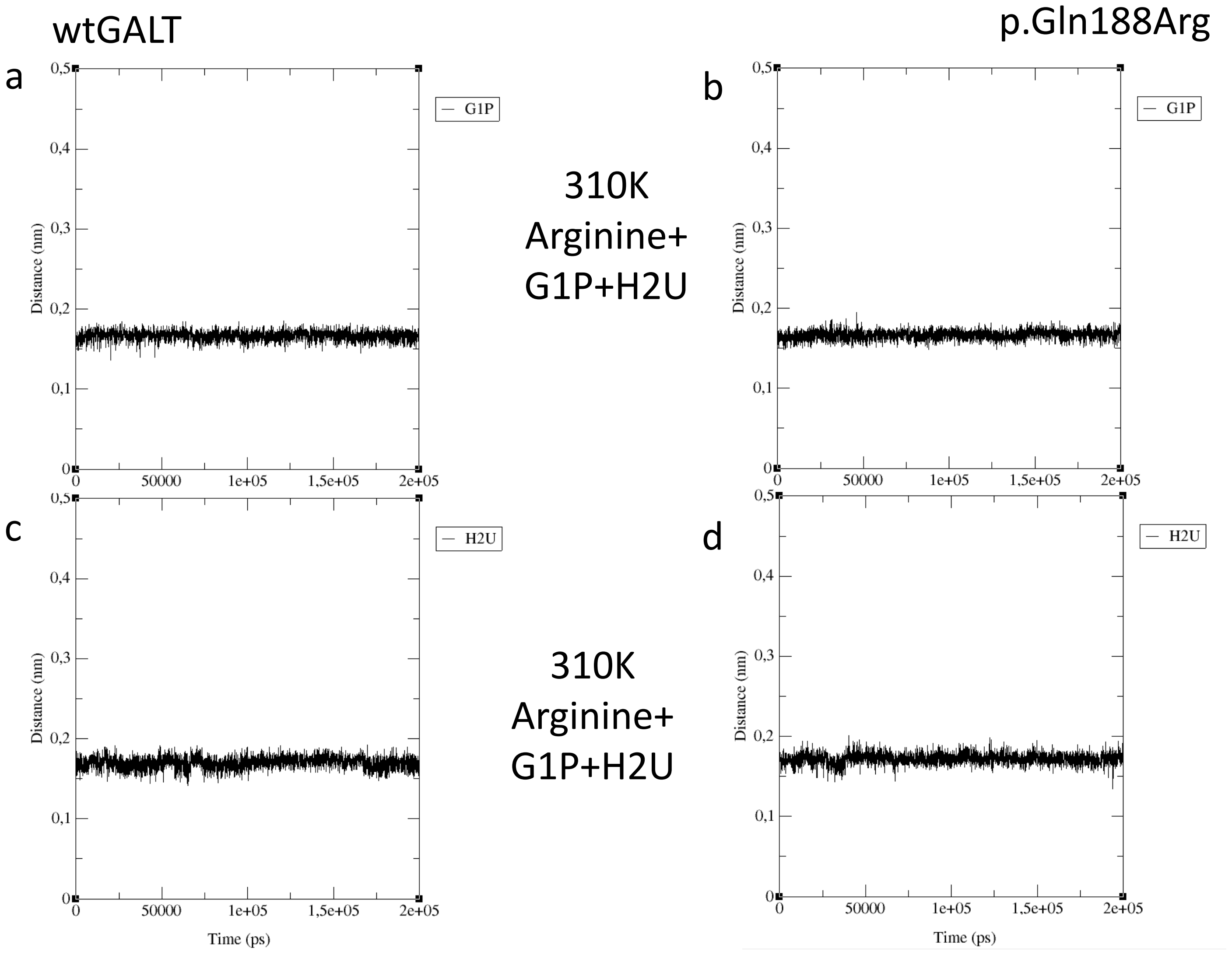

a): pair distance of G1P in the simulation wtGALT+G1P+H2U+arginine; b) pair distance of G1P in the simulation p.Gln188Arg+G1P+H2U+arginine; c): pair distance of H2U in the simulation wtGALT+G1P+H2U+arginine; b) pair distance of H2U in the simulation p.Gln188Arg+G1P+H2U+arginine

Figure S11
Radius of gyration
simulations with arginine in central cavity

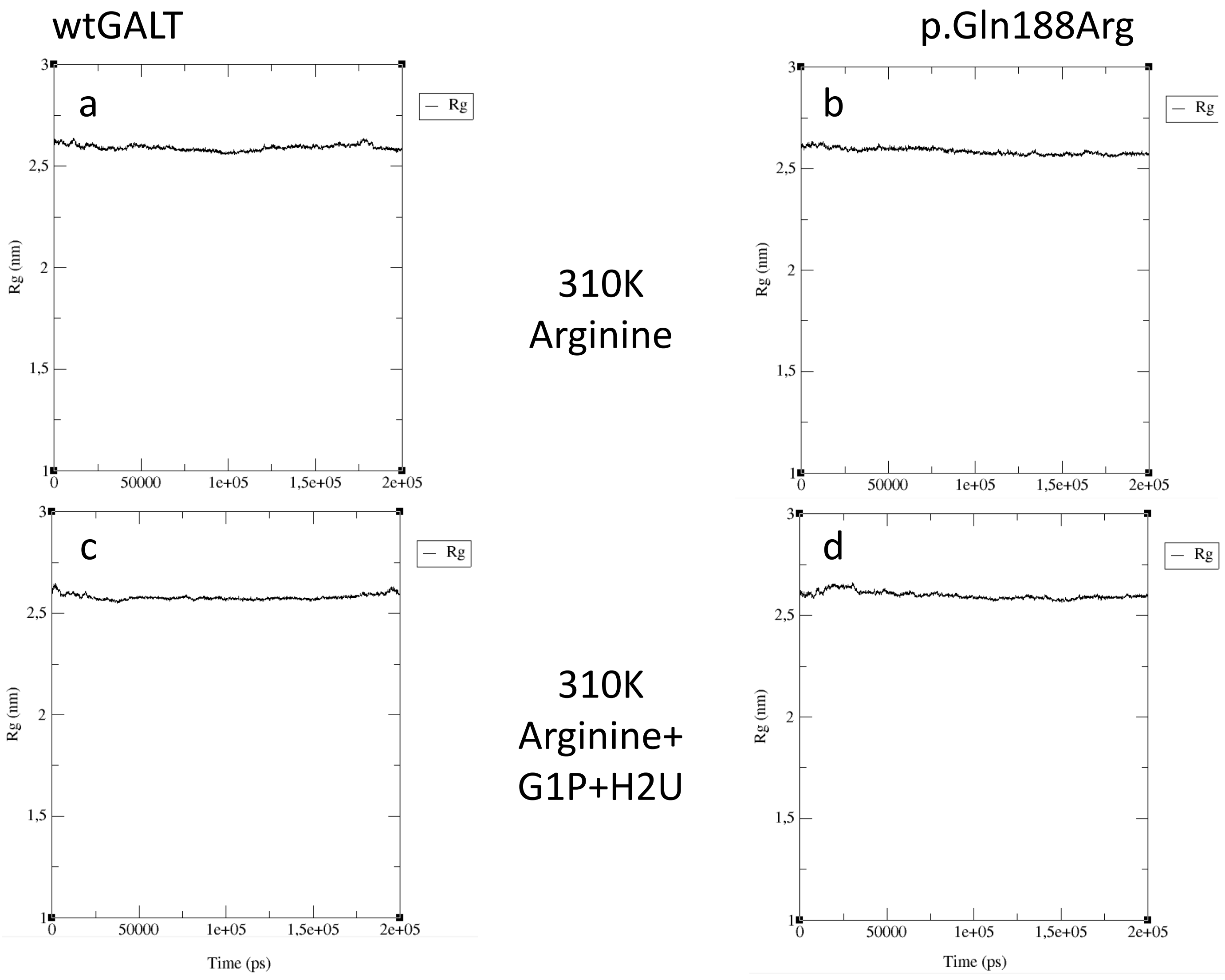

a): wtGALT+arginine; b): p.Gln188Arg+arginine; c) wtGALT+G1P+H2U+arginine; d) p.Gln188Arg+G1P+H2U+arginine.

Figure S12
SASA
simulations with arginine in the central cavity

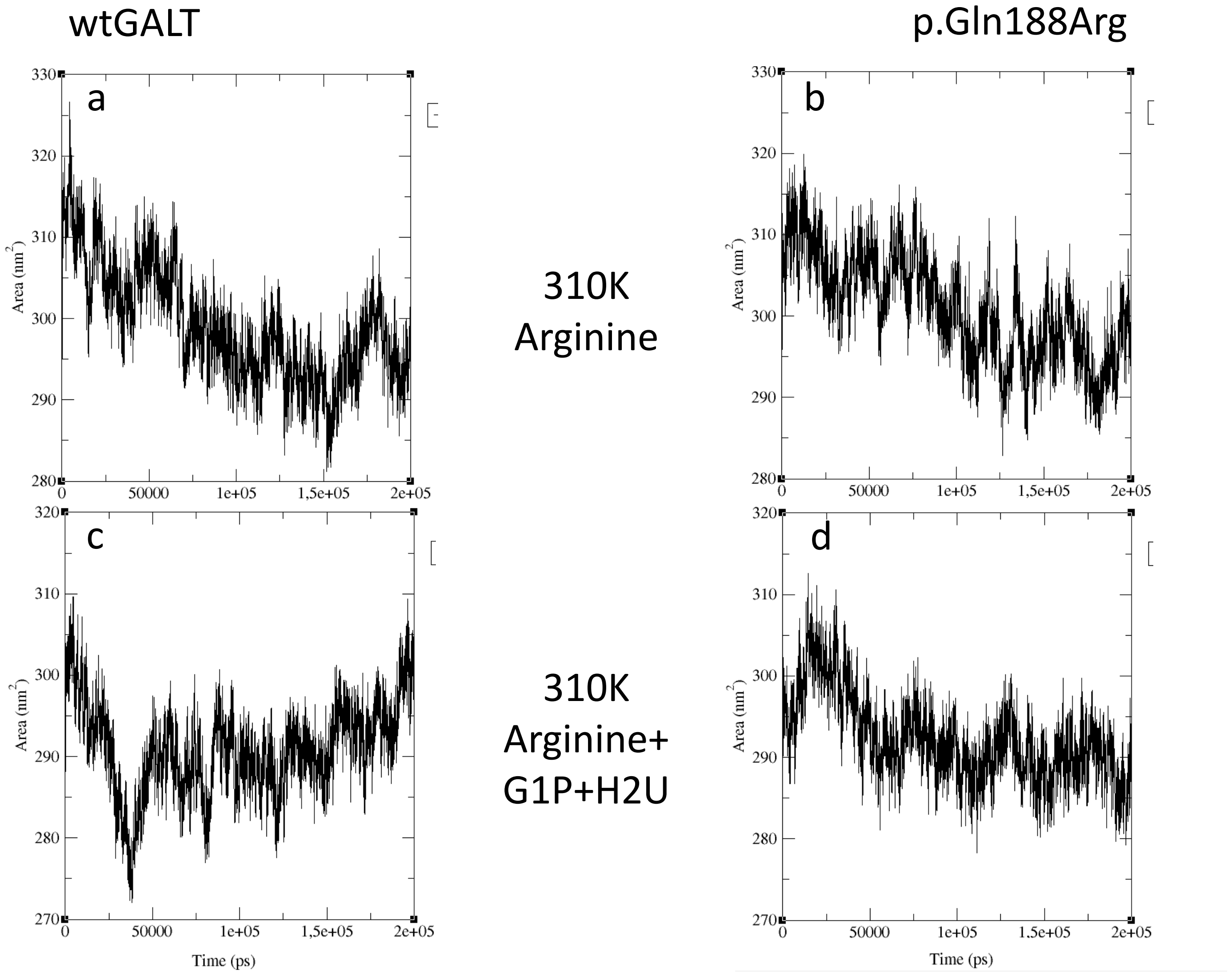

a): wtGALT+arginine; b): p.Gln188Arg+arginine; c) wtGALT+G1P+H2U+arginine; d) p.Gln188Arg+G1P+H2U+arginine.
