# Supplementary material for: Simulation of the Interactions of Arginine with Wild-Type GALT Enzyme and the Classic Galactosemia-Related Mutant p.Q188R by a Computational Approach
Source: Molecules. 2021 Oct 7;26(19):6061. doi: 10.3390/molecules26196061 (PMC8513022; doi:10.3390/molecules26196061)
Supplement: Supplementary file 1 [file molecules-26-06061-s001.zip › supplFiles-siArg_sito+cavita-rev.pdf]

Supplementary File 1
Quality check for wtGALT + Arg (active site)

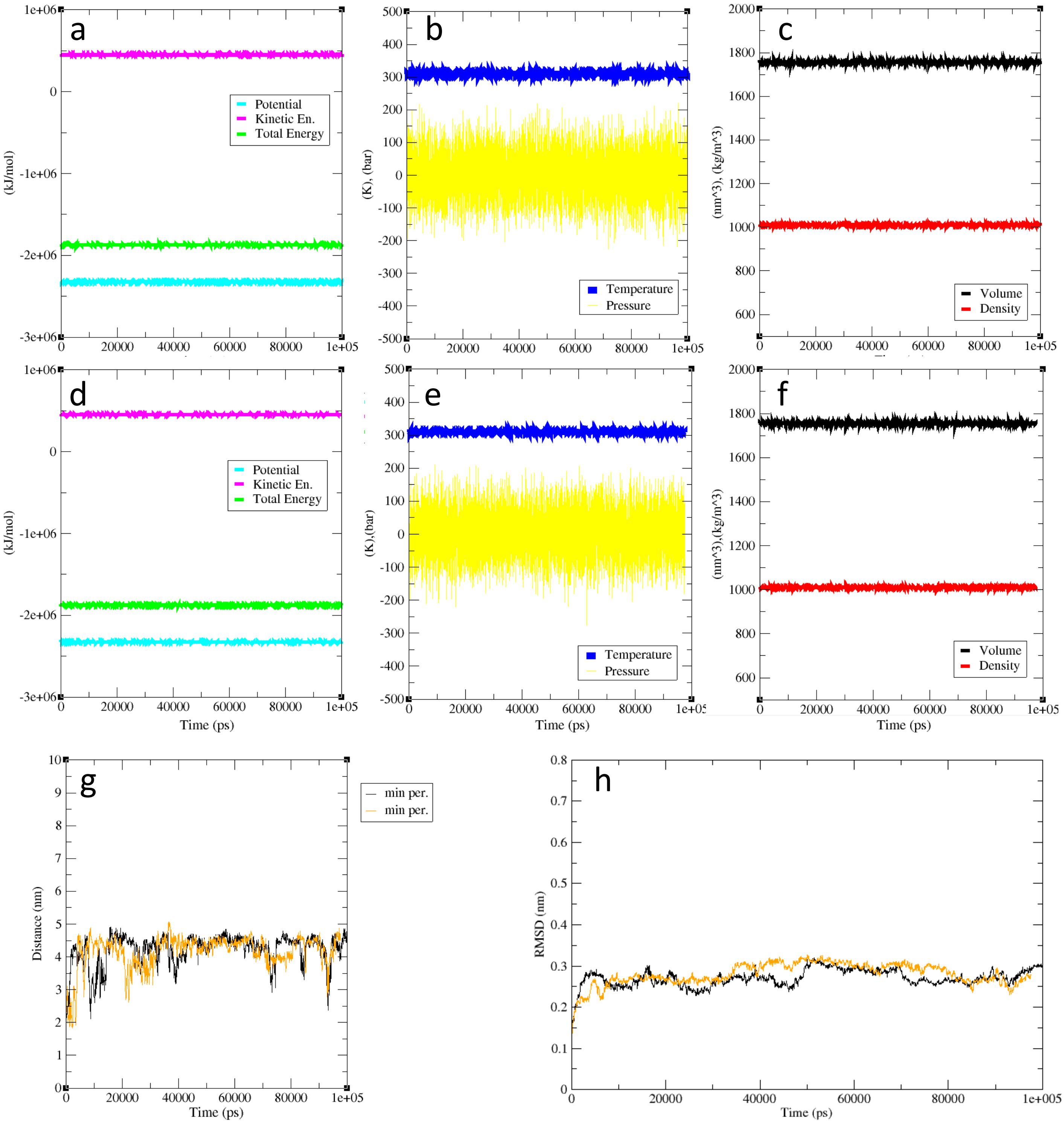

Legend: a: evolution of potential, kinetic and total energy of the first simulation; b: evolution of temperature and pressure of the first simulation; c: evolution of volume and density of the first simulation; d: evolution of potential, kinetic and total energy of the replica; e: evolution of temperature and pressure of the replica; f: evolution of volume and density of the replica; d: variation of minimum distance between periodic images of the two simulations; e: root mean square deviation (RMSD) of atom distances of the two simulations. Black line: first simulation; orange line: replica.

Supplementary File 2
Quality check for wtGALT + G1P + Arg (active site)

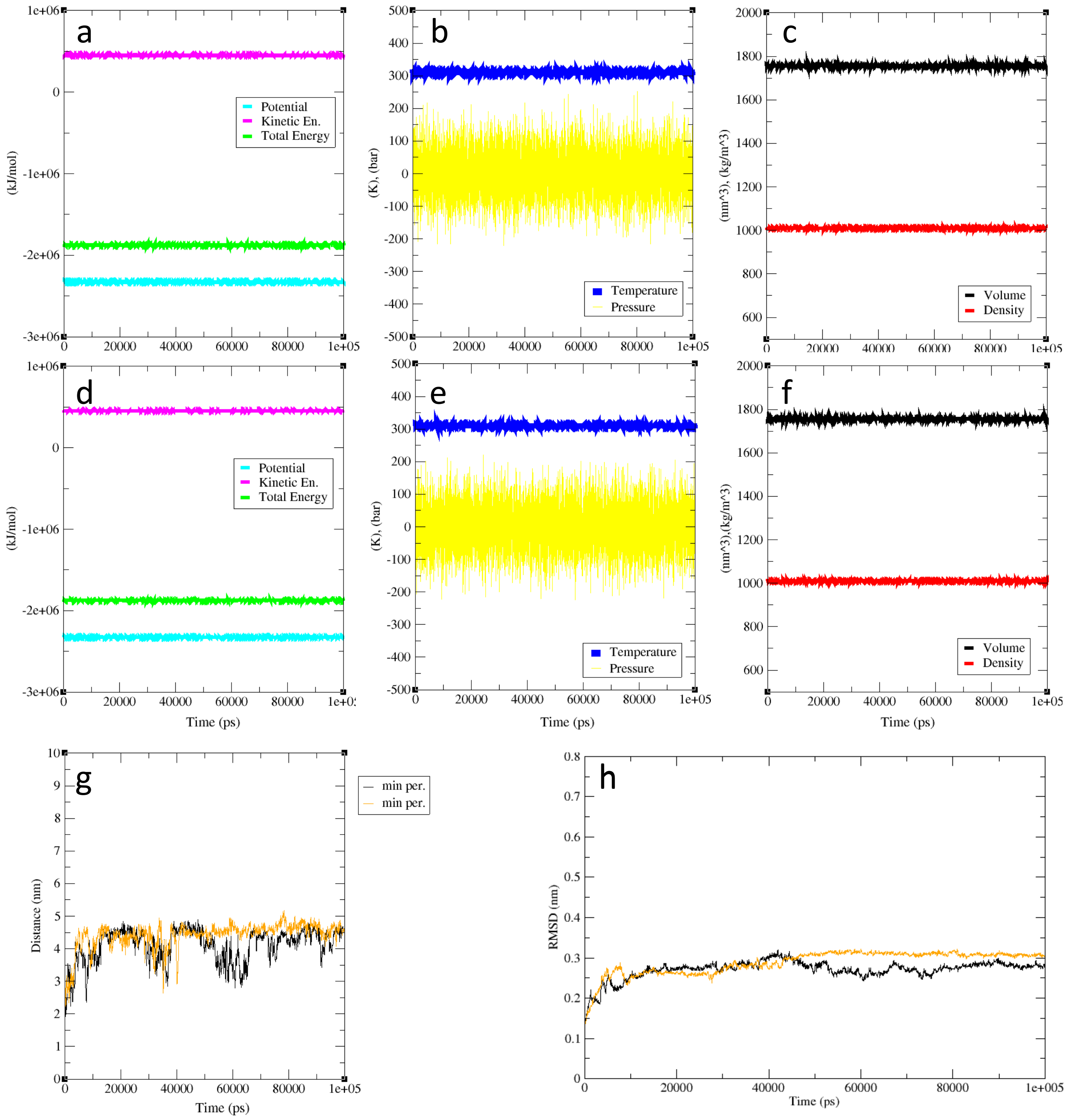

Legend: a: evolution of potential, kinetic and total energy of the first simulation; b: evolution of temperature and pressure of the first simulation; c: evolution of volume and density of the first simulation; d: evolution of potential, kinetic and total energy of the replica; e: evolution of temperature and pressure of the replica; f: evolution of volume and density of the replica; d: variation of minimum distance between periodic images of the two simulations; e: root mean square deviation (RMSD) of atom distances of the two simulations. Black line: first simulation; orange line: replica.

Supplementary File 3
Quality check for wtGALT + H2U + Arg (active site)

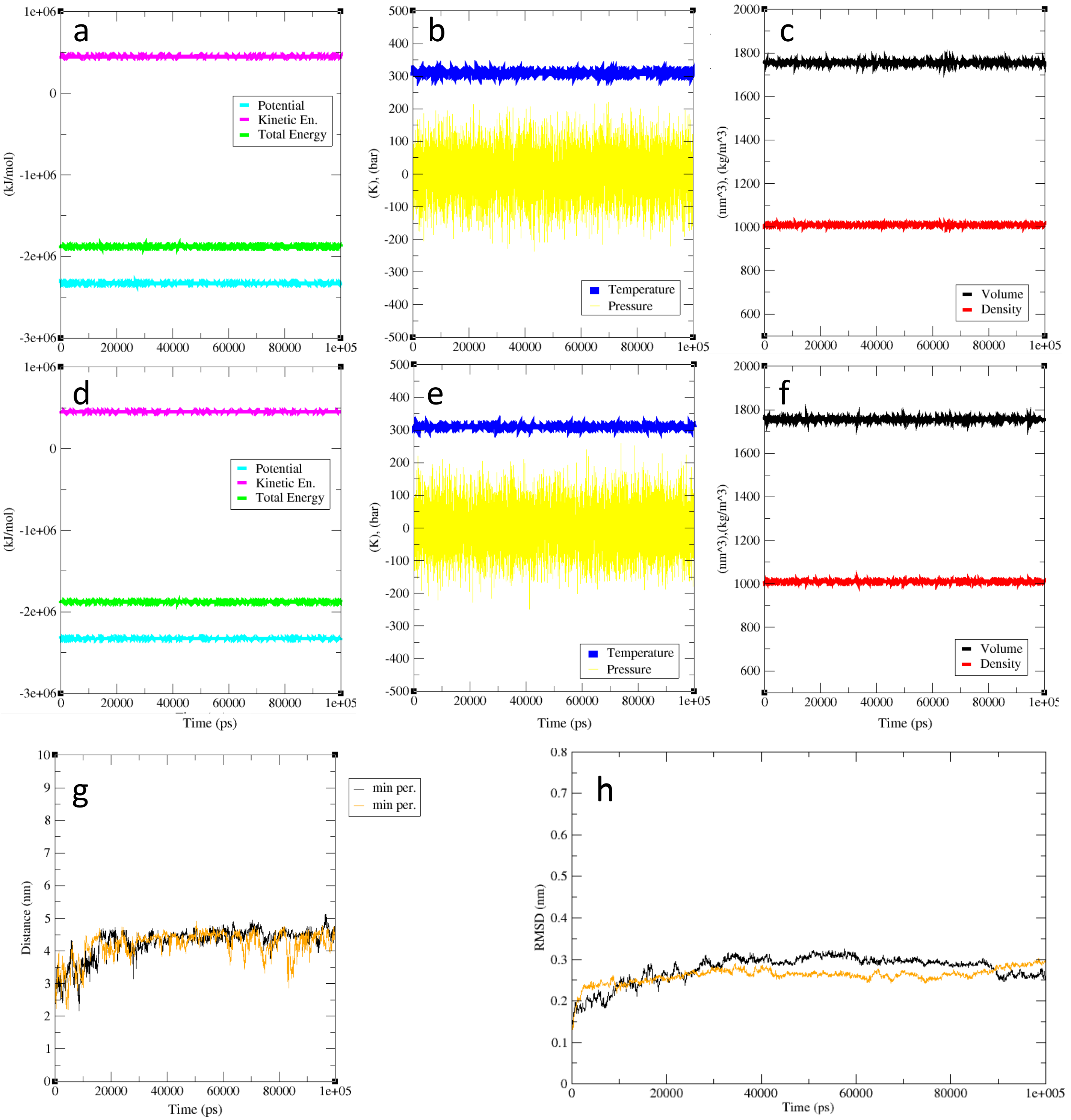

Legend: a: evolution of potential, kinetic and total energy of the first simulation; b: evolution of temperature and pressure of the first simulation; c: evolution of volume and density of the first simulation; d: evolution of potential, kinetic and total energy of the replica; e: evolution of temperature and pressure of the replica; f: evolution of volume and density of the replica; d: variation of minimum distance between periodic images of the two simulations; e: root mean square deviation (RMSD) of atom distances of the two simulations. Black line: first simulation; orange line: replica.

Supplementary File 4
Quality check for p.Gln188Arg + Arg (active site)

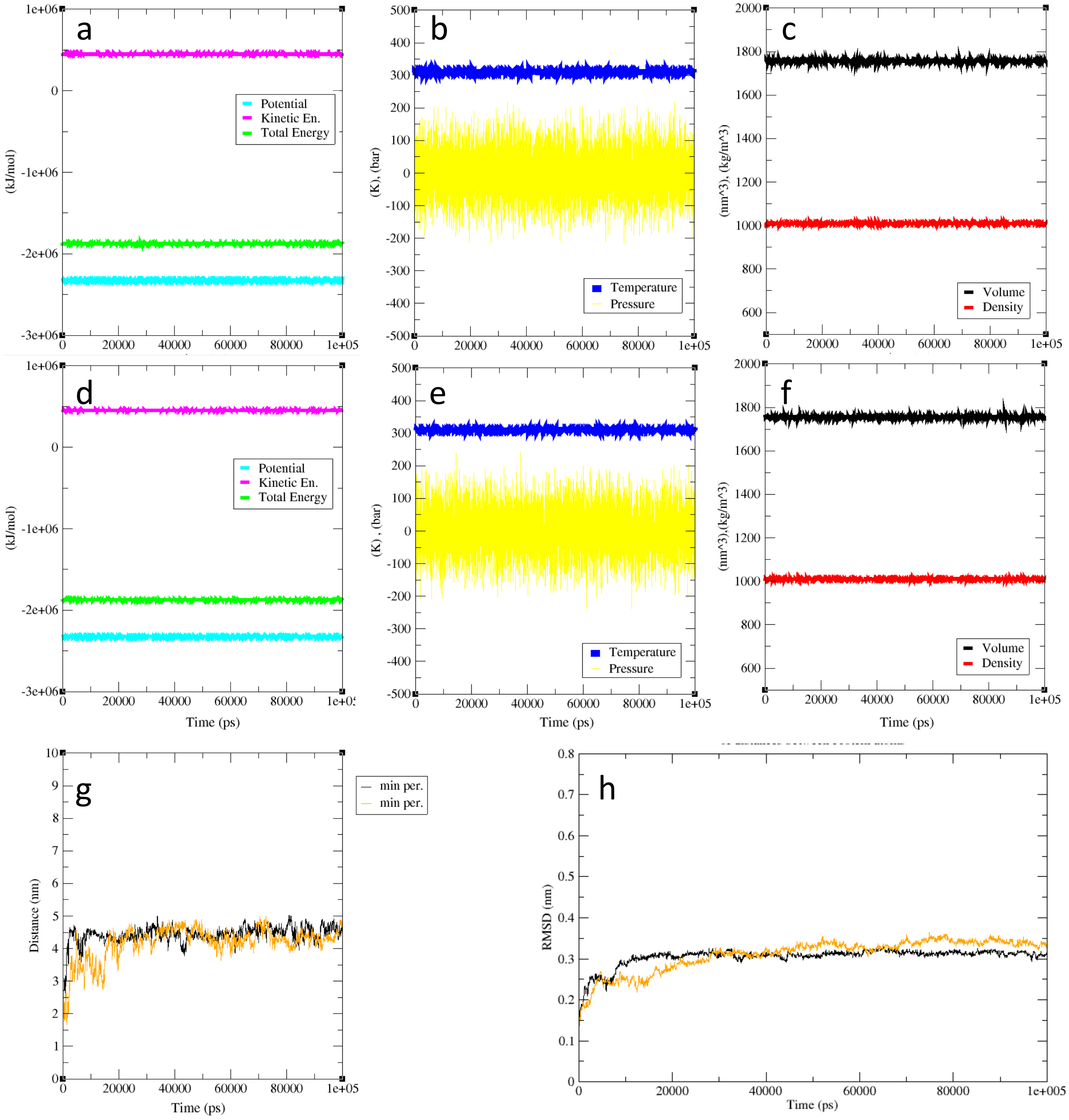

Legend: a: evolution of potential, kinetic and total energy of the first simulation; b: evolution of temperature and pressure of the first simulation; c: evolution of volume and density of the first simulation; d: evolution of potential, kinetic and total energy of the replica; e: evolution of temperature and pressure of the replica; f: evolution of volume and density of the replica; d: variation of minimum distance between periodic images of the two simulations; e: root mean square deviation (RMSD) of atom distances of the two simulations. Black line: first simulation; orange line: replica.

Supplementary File 5
Quality check for p.Gln188Arg + G1P + Arg (active site)

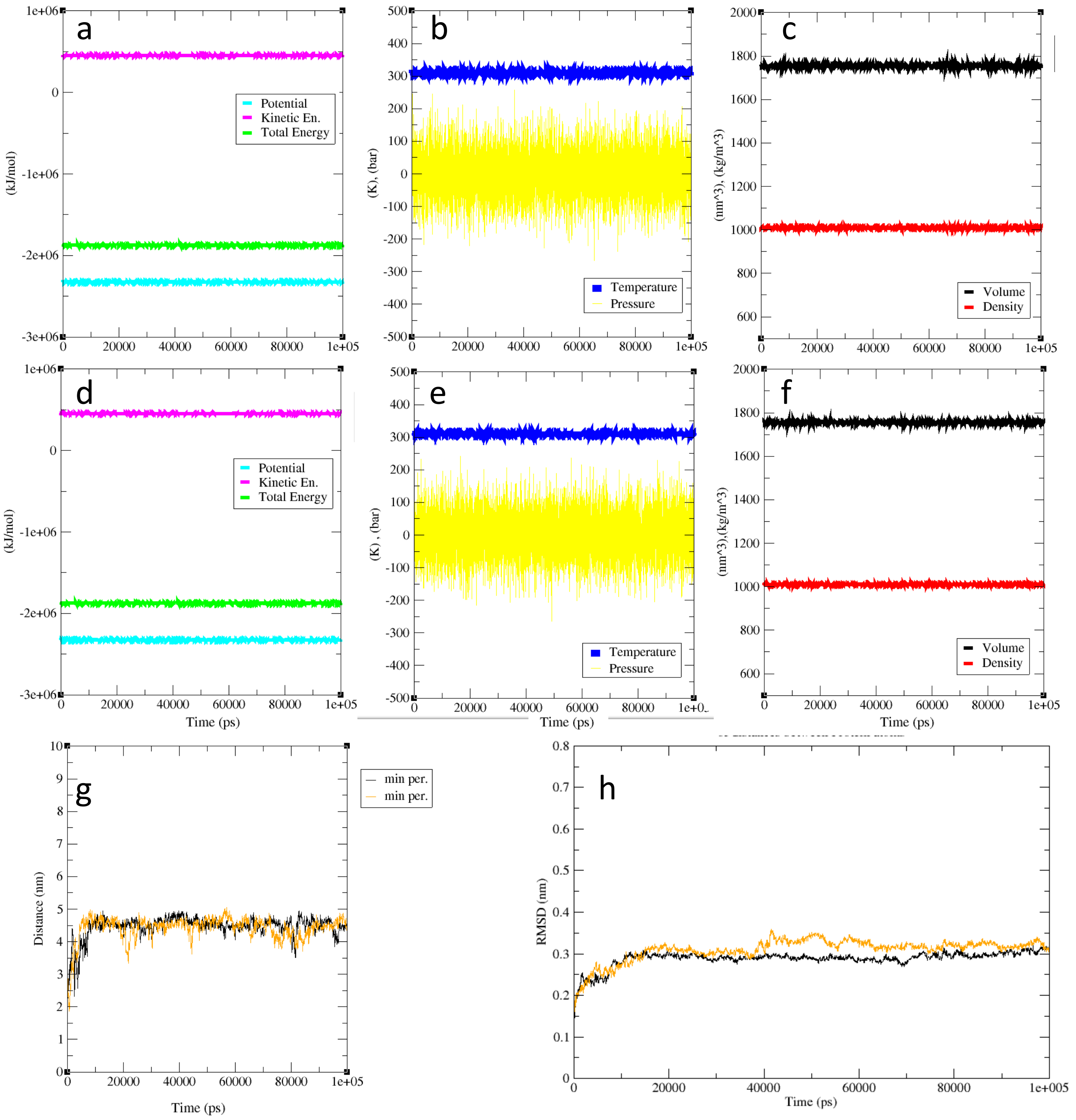

Legend: a: evolution of potential, kinetic and total energy of the first simulation; b: evolution of temperature and pressure of the first simulation; c: evolution of volume and density of the first simulation; d: evolution of potential, kinetic and total energy of the replica; e: evolution of temperature and pressure of the replica; f: evolution of volume and density of the replica; d: variation of minimum distance between periodic images of the two simulations; e: root mean square deviation (RMSD) of atom distances of the two simulations. Black line: first simulation; orange line: replica.

Supplementary File 6
Quality check for p.Gln188Arg + H2U + Arg (active site)

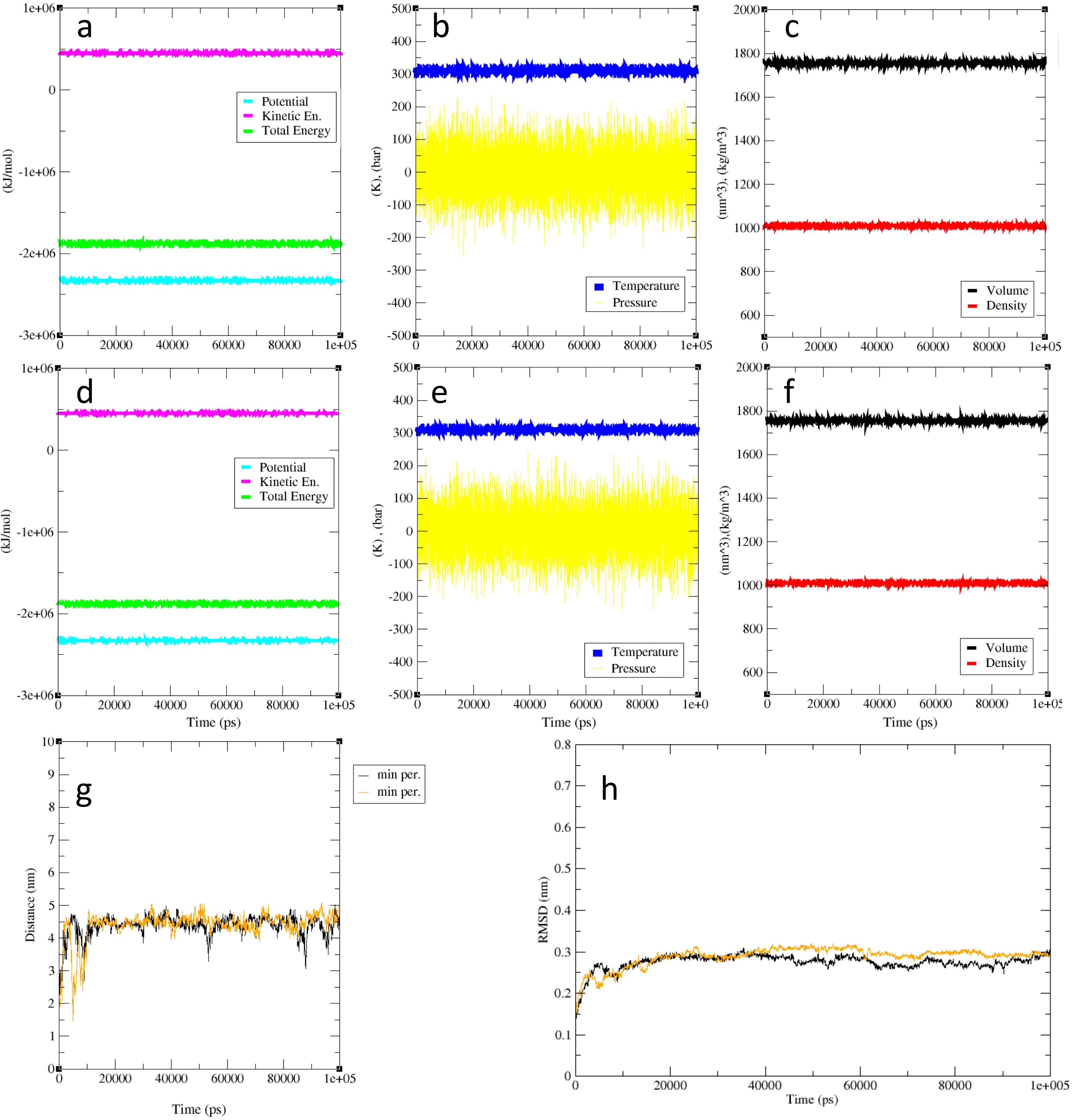

Legend: a: evolution of potential, kinetic and total energy of the first simulation; b: evolution of temperature and pressure of the first simulation; c: evolution of volume and density of the first simulation; d: evolution of potential, kinetic and total energy of the replica; e: evolution of temperature and pressure of the replica; f: evolution of volume and density of the replica; d: variation of minimum distance between periodic images of the two simulations; e: root mean square deviation (RMSD) of atom distances of the two simulations. Black line: first simulation; orange line: replica.

Supplementary File 7
Quality check for wtGALT + Arg (central cavity)

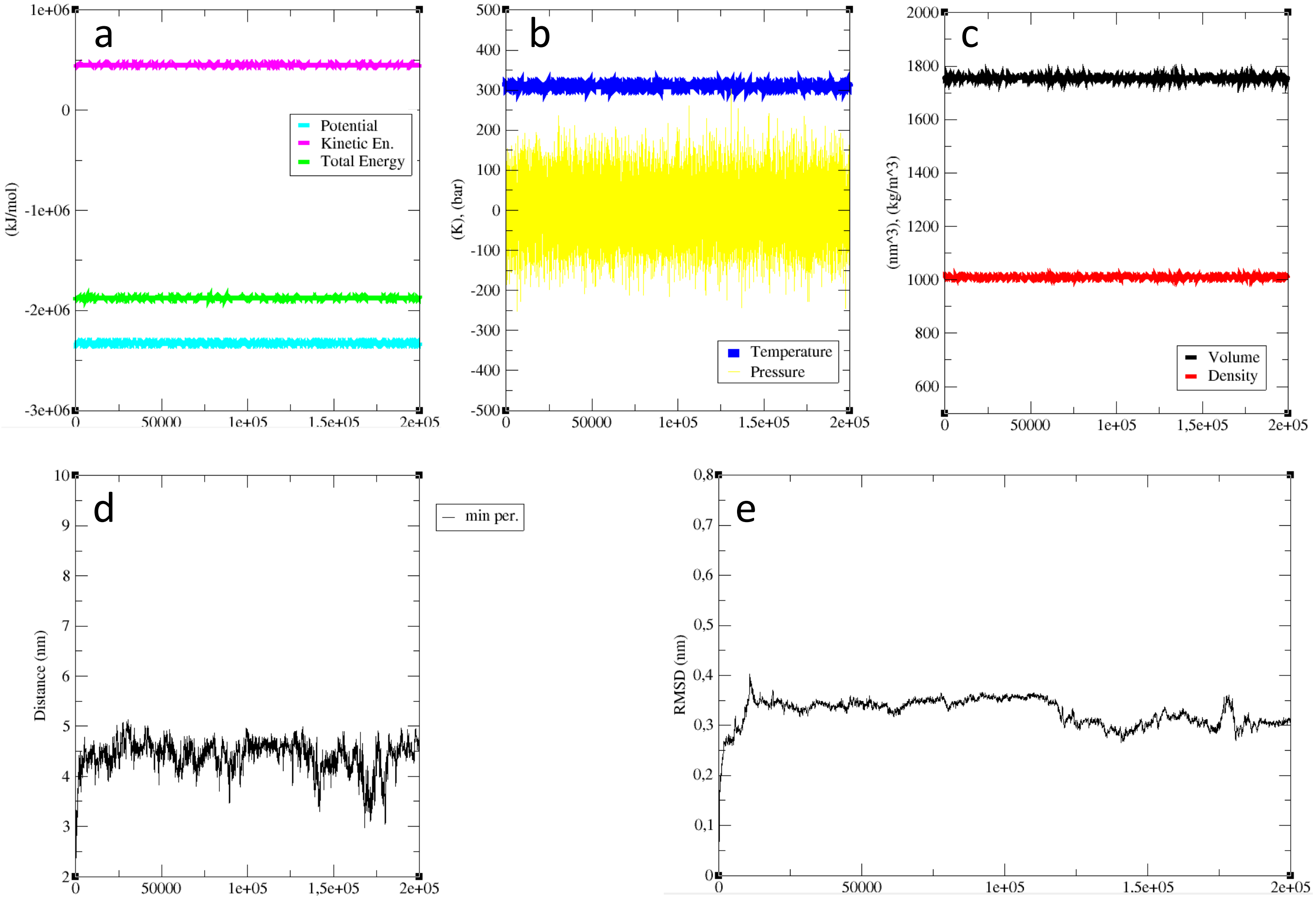

Legend: a: evolution of potential, kinetic and total energy; b: evolution of temperature and pressure; c: evolution of volume and density; d: variation of minimum distance between periodic images; e: root mean square deviation (RMSD) of atom distances.

Supplementary File 8
Quality check for wtGALT + ligands + Arg (central cavity)

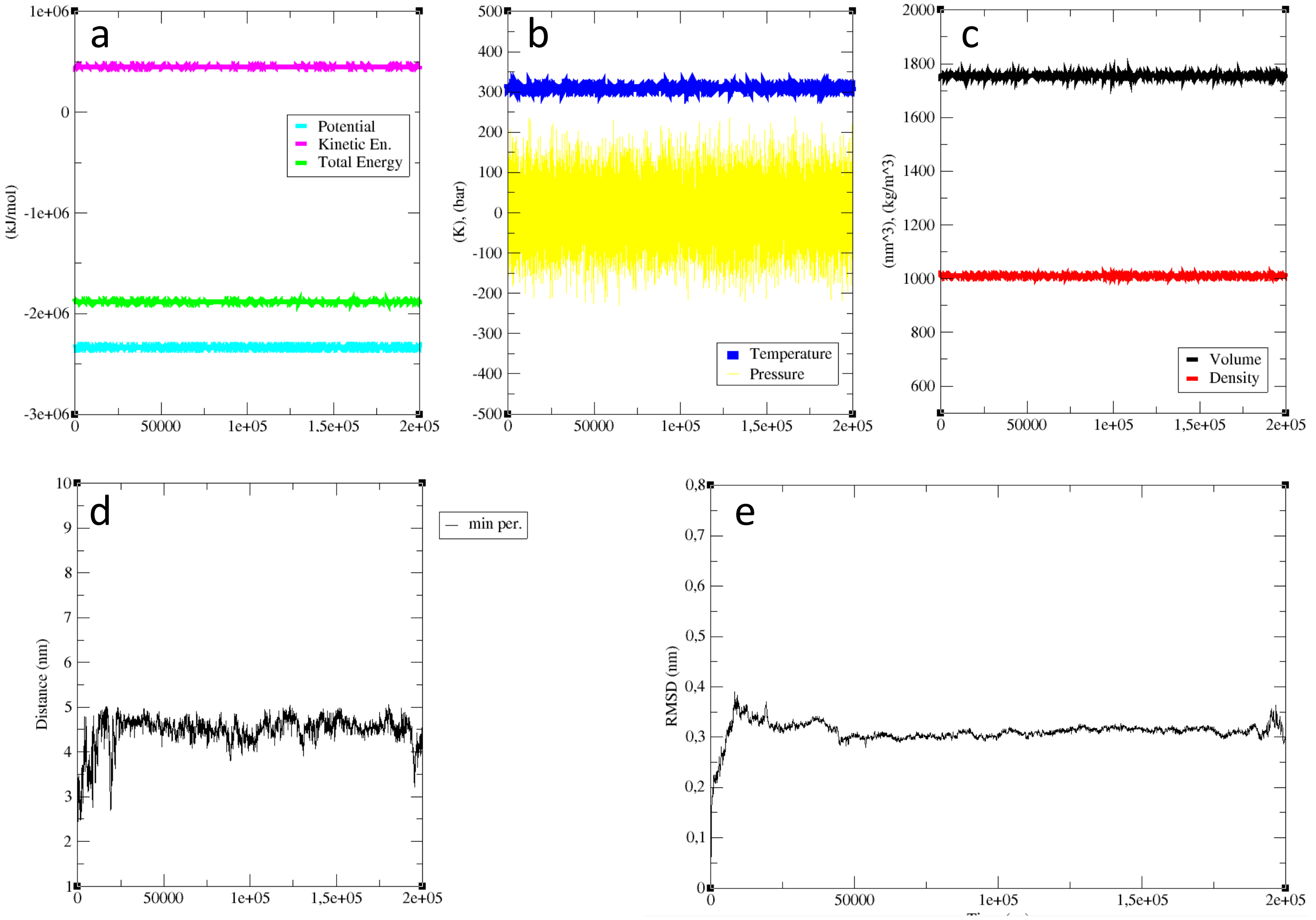

Legend: a: evolution of potential, kinetic and total energy; b: evolution of temperature and pressure; c: evolution of volume and density; d: variation of minimum distance between periodic images; e: root mean square deviation (RMSD) of atom distances.

Supplementary File 9
Quality check for p.Gln188Arg + Arg (central cavity)

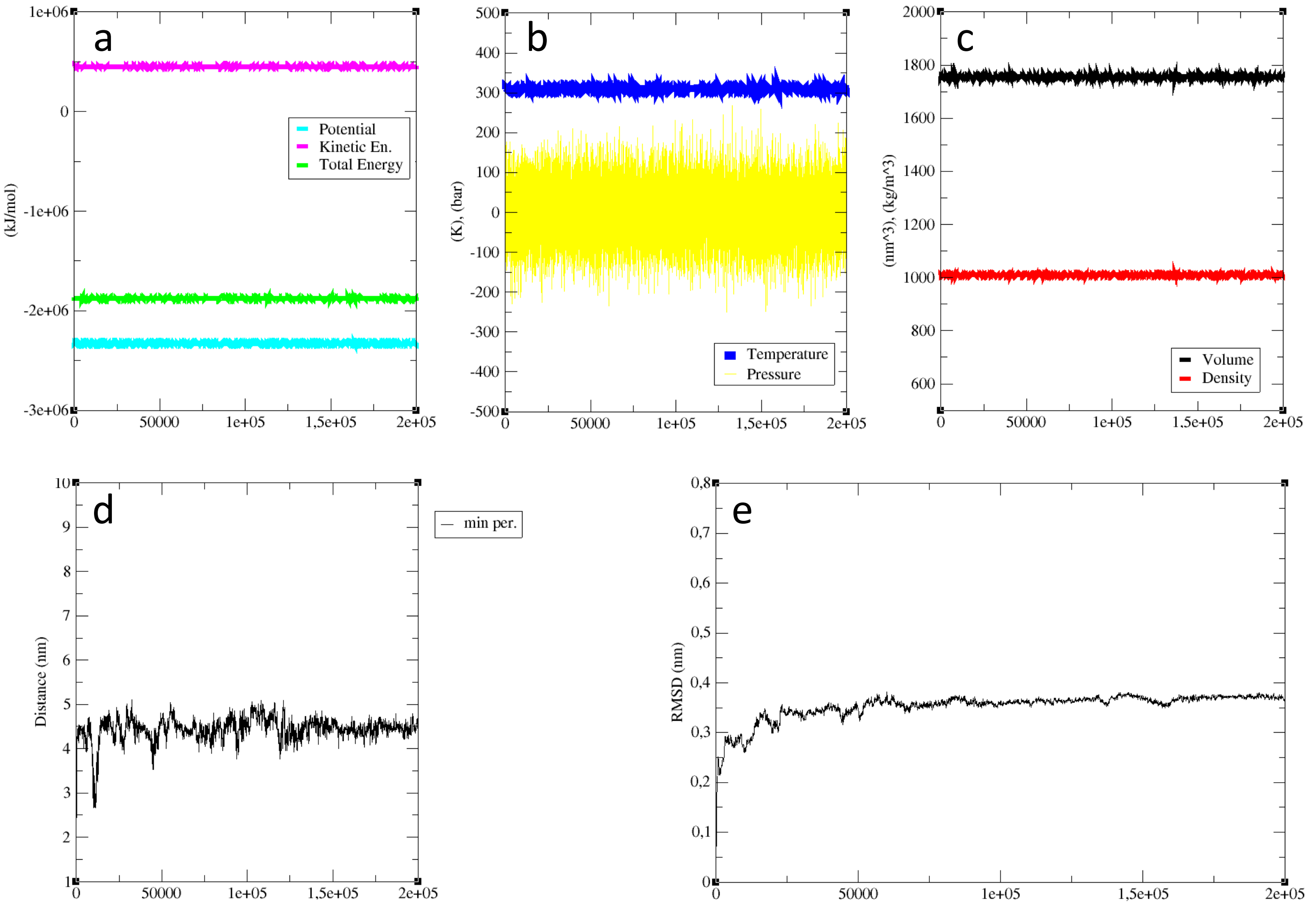

Legend: a: evolution of potential, kinetic and total energy; b: evolution of temperature and pressure; c: evolution of volume and density; d: variation of minimum distance between periodic images; e: root mean square deviation (RMSD) of atom distances.

Supplementary File 10
Quality check for p.Gln188Arg + ligands + Arg (central cavity)

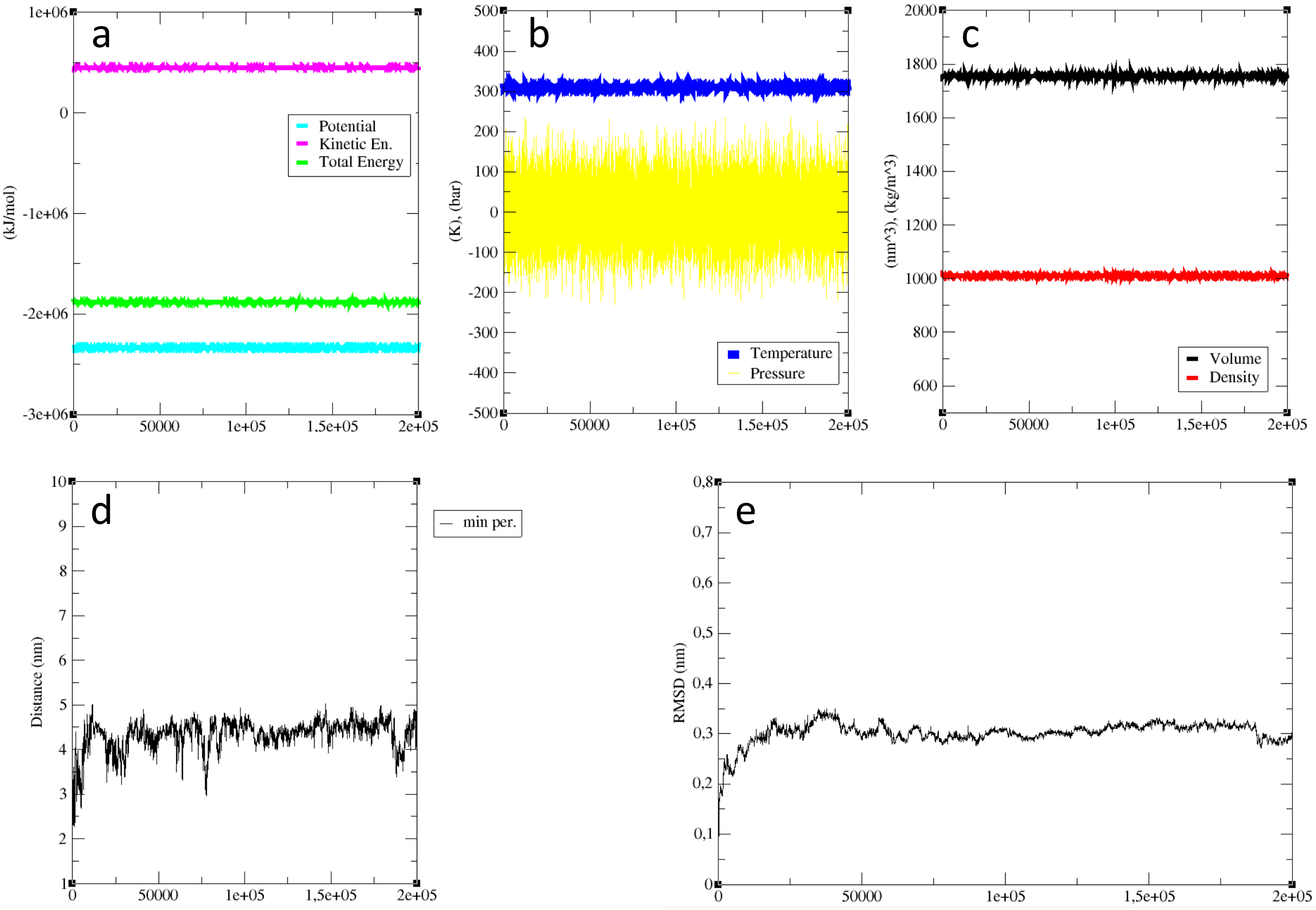

Legend: a: evolution of potential, kinetic and total energy; b: evolution of temperature and pressure; c: evolution of volume and density; d: variation of minimum distance between periodic images; e: root mean square deviation (RMSD) of atom distances.
